# Nutrients from salmon parents alter selection pressures on their offspring

**DOI:** 10.1111/ele.12894

**Published:** 2017-12-15

**Authors:** Sonya K. Auer, Graeme J. Anderson, Simon McKelvey, Ronald D. Bassar, Darryl McLennan, John D. Armstrong, Keith H. Nislow, Helen K. Downie, Lynn McKelvey, Thomas A.J. Morgan, Karine Salin, Danielle L. Orrell, Alice Gauthey, Thomas C. Reid, Neil B. Metcalfe

**Affiliations:** ^1^ Institute of Biodiversity, Animal Health and Comparative Medicine University of Glasgow Glasgow G12 8QQ UK; ^2^ Cromarty Firth Fishery Trust Inverness IV2 3HF UK; ^3^ Department of Biology Williams College Williamstown MA 01267 USA; ^4^ Freshwater Fisheries Laboratory Marine Scotland–Science Pitlochry PH16 5LB UK; ^5^ USDA Forest Service Northern Research Station Amherst MA 01003 USA; ^6^ Université Pierre et Marie Curie Paris 75005 France

**Keywords:** Eco‐evolutionary dynamics, eco‐evolutionary feedbacks, natural selection, niche construction, *Salmo salar*, selection differential, selection gradient

## Abstract

Organisms can modify their surrounding environment, but whether these changes are large enough to feed back and alter their evolutionary trajectories is not well understood, particularly in wild populations. Here we show that nutrient pulses from decomposing Atlantic salmon (*Salmo salar*) parents alter selection pressures on their offspring with important consequences for their phenotypic and genetic diversity. We found a strong survival advantage to larger eggs and faster juvenile metabolic rates in streams lacking carcasses but not in streams containing this parental nutrient input. Differences in selection intensities led to significant phenotypic divergence in these two traits among stream types. Stronger selection in streams with low parental nutrient input also decreased the number of surviving families compared to streams with high parental nutrient levels. Observed effects of parent‐derived nutrients on selection pressures provide experimental evidence for key components of eco‐evolutionary feedbacks in wild populations.

## Introduction

Ecological conditions set the stage for evolutionary change. However, there is increasing evidence that ecological and evolutionary processes can occur on similar timescales scales, thereby opening up the potential for dynamic interactions between them in contemporary time (Post & Palkovacs 2009; Hendry 2017). Organisms can alter their surrounding environment via processes such as habitat modification, nutrient cycling and consumption of resources (Post & Palkovacs 2009; Matthews *et al*. 2014). These modifications may, in turn, influence selection pressures that feed back to alter their own evolutionary trajectory (Post & Palkovacs 2009; Matthews *et al*. 2014; Hendry 2017). Reciprocal interactions between ecological and evolutionary processes have been demonstrated under laboratory conditions (Post & Palkovacs 2009; Hendry 2017). For example, rapid evolution of prey traits in chemostats has ecological impacts on predators which then feed back to alter prey gene frequencies (Yoshida *et al*. 2003, 2004). However, direct experimental evaluation of these eco‐evolutionary feedbacks in wild populations is currently limited.

Here we examine whether nutrient pulses from decomposing Atlantic salmon (*Salmo salar*) parents alter selection pressures on their offspring. Salmon and other anadromous fishes convey limiting nutrients to inland waters when they return from the sea to spawn (Naiman *et al*. 2002; Jonsson & Jonsson 2003; Walters *et al*. 2009; Flecker *et al*. 2010; Childress *et al*. 2014). Marine‐derived nutrient subsidies from their waste products, gametes and post‐spawning carcasses can, in turn, have positive impacts on the macroinvertebrate prey and subsequent density and biomass of their offspring as well as the overall productivity of the freshwater and surrounding terrestrial environments (Naiman *et al*. 2002; Nislow *et al*. 2004; Williams *et al*. 2009; Moore *et al*. 2011; Guyette *et al*. 2013; Childress *et al*. 2014). By increasing environmental productivity, these parental nutrients may also shape the selective environment experienced by their offspring but their evolutionary consequences have remained unstudied.

Parental nutrient levels and subsequent food availability may alter selection on organismal traits related to energy requirements and expenditure. Egg size and juvenile metabolic rate are both heritable traits with important consequences for early growth and survival that are thought to depend on food availability (Einum *et al*. 2004; Metcalfe *et al*. 2016). Theory (Einum *et al*. 2004) and empirical studies (Hutchings 1991; Einum *et al*. 2004; Rollinson & Hutchings 2013a) suggest that a larger egg size, with consequent higher maternal nutritional investment, is most advantageous under competitive conditions where food availability and subsequent growth opportunities are limited. In addition, laboratory and mesocosm studies suggest that a high metabolic rate is beneficial for fitness components such as growth and survival when food resources are high but disadvantageous when resources are low (Bochdansky *et al*. 2005; Burton *et al*. 2011; Auer *et al*. 2015). However, individuals with higher metabolic rates also tend to be dominant and secure preferential access to food (Reid *et al*. 2011; Metcalfe *et al*. 2016), potentially resulting in stronger selection for high metabolic rates in energy‐limited environments. Decreased parental nutrient input and associated decreases in prey availability may therefore select for larger egg size, but either lower or higher metabolic rates depending on the relative importance of energy conservation vs. priority of access to food. However, these hypotheses have not yet been examined experimentally in the wild.

We examined survival selection on egg size and juvenile metabolic rates in wild Atlantic salmon (*Salmo salar*) populations subjected to manipulated levels of marine‐derived parental nutrients in replicate tributary streams of the River Conon, northern Scotland (Fig. S1, Table S1). Nutrient levels in this system are currently reduced due to a net export of nutrients caused by reduced nutrient input (import via eggs but not adults) relative to nutrient export via emigration of juveniles stocked as eggs (Nislow *et al*. 2004). We selected 300 m^2^ reaches in 10 study streams with similar habitat and added analogue carcasses in the form of dried hatchery salmon pellets (Wipfli *et al*. 2004) to these reaches in five of the selected streams to increase nutrients to levels traditionally supported by the death and decomposition of post‐spawning parents (‘high nutrient’ streams hereafter). The remaining five streams served as reference sites (‘low nutrient’ streams hereafter). We then planted eggs from full‐sibling salmon families in equal distribution and density across these same reaches in all 10 study streams. The impact of parental nutrients was assessed first by measuring the biomass and abundance of macroinvertebrates in the size range consumed by juvenile salmon. We measured egg mass in a subset of eggs from each family and measured standard (= minimum) and maximum metabolic rates (Auer *et al*. 2017) in juveniles reared from a second subset of eggs retained in the laboratory. We then combined these family level estimates with measurements of family level egg‐to‐juvenile survival over a four‐month period in the wild to compare selection gradients for egg size and metabolic rates and subsequent phenotypic and genetic diversity between streams with low and high parental nutrient levels.

## Methods

### Study sites

Study sites were small headwater streams surrounded primarily by open moorland and some pastureland in northern Scotland (Nislow *et al*. 2004). In each stream, a 300 m^2^ section was selected for the study area (75–100 m in length depending on stream wet width; see Table S1). Streams drained into the Rivers Blackwater, Bran and Meig that form part of the greater River Conon catchment (Fig. S1, Table S1). All four of these rivers have been dammed to varying extents as part of a larger hydroelectric power scheme set up during the mid‐20th century (Williams 2007). Since that time a small number of returning adult salmon successfully navigate the dam lifts and ladders each year, but none migrate far enough upstream to spawn in these study areas (Gowans *et al*. 2003). Atlantic salmon (stocked as eggs) and resident brown trout (*Salmo trutta*) are the dominant fish species in the system.

### *In vitro* fertilisation and hatchery rearing of eggs

Fifty‐four full sibling families were created within a three‐day period in the first week of December 2015. Crosses were made using eggs and sperm from wild returning adult salmon caught in a fish trap at a dam on the River Blackwater (Fig. S1). Adults were fish previously stocked as eggs or unfed fry in the headwaters upstream of the dam where they spent 2‐4 years (mean ± 1 SE: 2.45 ± 0.03 years confirmed by scalimetry for females) before heading to sea. Crosses were random with respect to male age, but we controlled for maternal effects on egg mass by randomly selecting a subset of 30 families with the most frequent maternal life history, where the female had spent only one winter at sea (confirmed by scalimetry). A small portion of the adipose fin was clipped and preserved in 100% ethanol for later DNA analysis (see below). A sample of eggs from each clutch was preserved in a 5% buffered formalin solution (Fleming & Ng 1987) for later determination of mean egg mass per family. The remaining fertilised eggs were then transported to a nearby hatchery where they were reared in family specific trays but under identical water and temperature conditions.

### Planting out eggs and analogue carcasses

On reaching the eyed stage, 100 eggs from each of the 30 families were collected and transferred to a tray for each of the 10 streams. Eggs were then planted out in each stream in late February/early March 2016 (Table S1). Each 300 m^2^ stream section received a total of 3000 eggs (100 from each of the 30 families), equating to a density of 10 eggs m^−2^ and matching typical spawning densities (Fleming 1996). Eggs were deposited in eight artificial nests, as detailed in (McLennan *et al*. 2016), distributed at equidistant locations between the top and bottom of each experimental reach (*n* = 350 eggs per nest). The remaining 200 eggs were buried beneath the gravel in two Vibert boxes, one at the top and bottom of each experimental reach (*n* = 100 eggs per box), to assess hatching success. The Vibert boxes were empty when recovered in late May/early June 2016 during the time of macroinvertebrate sampling (see below), indicating that no egg mortalities had occurred after their deposition in the stream.

Analogue carcasses were added to five of the study streams (selected randomly) at the time of egg planting. Analogue carcasses were composed of dried hatchery salmon pellets (Coral 2000 + 40PAX B12 60% marine‐derived fish‐based nutrients, Skretting, Invergordon UK) that have a similar nutritional content and decay rate to salmon carcasses (Pearsons *et al*. 2007). Each of the five streams received five 3‐kg carcass analogues to simulate the death and decomposition of approximately 25 adult salmon carcasses, an amount similar to or less than that used in other nutrient supplementation experiments in nutrient poor Atlantic salmon streams (Williams *et al*. 2009; Guyette *et al*. 2013, 2014). Carcass analogues were buried at equidistant points along each experimental reach. HOBO temperature data loggers (Onset Computer Corporation, Bourne MA, USA) were placed in each stream at the time of egg deposition and programmed to record the temperature every 4 h.

### Metabolic rates

Metabolic rates were measured in a subset of siblings from each of the 30 focal families that were transferred during the egg stage in late February 2016 and raised to the fry stage in a laboratory at the University of Glasgow. Metabolic rates were measured at 12°C over a 10‐day period during the last 2 weeks of June 2016, when fry were approximately 2 months old (*n* = 10 fry per family). Standard metabolic rate was measured over a 20 h period as the rate of oxygen consumption using continuous flow‐through respirometry, following methods described in (Auer *et al*. 2015). Maximum metabolic rate was then determined using an exhaustive chase protocol followed immediately by measurement of oxygen consumption during the recovery period using closed‐system respirometry, following Auer *et al*. (2015). After their metabolic rate measurements, fish were weighed (± 1.0 mg) and measured for body length (± 0.01 mm) under a mild anaesthetic (Benzocaine 40 mg L^−1^). Metabolic rates were then standardised to a common body size of 1 g. At the time of measurement, fry in the laboratory were on average slightly smaller but within the size range of their siblings captured roughly 3–4 weeks later in the field (mean fork length ± 1 SE: lab: 39.24 ± 0.17 mm, range 29.90–46.80; *n* = 300; field: 48.17 ± 0.20 mm, range 26.92–70.20; *n* = 1246). Measurements at 12°C approximated the mean daily water temperature in the field (range: 10.8 to 14.4°C) at that time (Fig. S3).

### Macroinvertebrate abundance and biomass

Macroinvertebrates were sampled from late May to early June 2016 by electro‐bugging (Taylor *et al*. 2001) three contiguous locations at each of three positions (0, 25 and 50 m up from the downstream limit of the experimental reach) in each of the 10 streams. Invertebrates were shocked (60‐s pulse) using a 500 W electrofishing backpack system (E‐fish ltd, Grange‐over‐Sands, UK; 350 V, 60 Hz and a 10% duty cycle) and trapped in a Surber sampler (250 μm filter; EFE and GB Nets, Totnes, UK) placed on the substrate directly downstream from the anode (Taylor *et al*. 2001). Macroinvertebrates from each of the three pulsed samples were preserved together in 70% ethanol, and later identified to the family level under a dissecting scope, counted to determine numerical abundance, and measured to the nearest 0.5 mm in length to determine their dry weight and total biomass (Bird & Prairie 1985). Biomass was determined using published length‐mass regressions for the relationship between dry mass and length for each taxonomic family (Table S3; Benke *et al*. 1999). Abundance and biomass were defined as catch per unit effort since the area upstream from the anode was not blocked off. Gape size limits the size of prey that juvenile salmon can consume (Wankowski 1979), so only macroinvertebrates equal to or smaller than 1 mm in width (the maximum size that could be ingested by first feeding juveniles; Wankowski 1979) were included in estimates of prey abundance and biomass. These included members of the Baetidae, Ephemerellidae, Chironomidae and Simuliidae families, all of which are prey items for juvenile salmon in this and other populations (Mills 1964; Maitland 1965).

### Recapture of wild juvenile salmon

Surviving wild fry were captured by electro‐fishing over a 2‐week period in July 2016 (Table S1). At each stream, at least two 12 m sections of riffle habitat within each of the experimental reaches were electro‐fished with three passes per section to estimate fish density. The rest of each experimental reach was also electro‐fished (generally 1 pass) to increase family level sample sizes needed to estimate survival. Fish were weighed (± 1.0 mg) and measured for body length (± 0.01 mm) under a mild anaesthetic (clove oil 20 ppm). In addition, a small portion of their anal fin was clipped and stored in ethanol for later parental assignment (see below). The density of salmon fry was estimated using Microfish (Van Deventer & Platts 1989). Small numbers of older year classes of juvenile salmon (immigrants from neighbouring stocked streams) and trout were also captured in nine of ten study streams (Table S1), but the densities (number captured per square metre area) of these parr did not differ among streams with low vs. high parental nutrients (*P *=* *0.309).

### Genotyping and parental assignment

Captured fry were genotyped and assigned to a specific focal family by commercial suppliers (Landcatch Natural Selection Ltd, Stirling, Scotland) using a panel of markers customised for internal use. DNA was extracted from the fin clips of all parental fish and recaptured offspring using an E‐Z 96 tissue DNA Tissue kit (Omega Bio‐Tek, Norcross, Georgia, USA) following the manufacturer's protocol. Genotyping was performed using a panel of 110 informative SNP markers scattered across the genome. Individual end point PCR assays were designed and developed (KASP TM technology, UK) for the genotyping of samples. Parentage assignment by exclusion was carried out blind to experimental treatments with the programme Vitassign 8.3 (Vandeputte *et al*. 2006) with some modifications to allow the analysis of more than 100 markers. Of the 1272 juvenile salmon captured, 1246 (98.0%) were uniquely assigned to one of the full sibling 30 families. Of those not assigned, 10 samples were crosses between two focal families likely caused by milt contamination. In addition, no samples were assigned to one focal family, whereas 16 samples were not assigned to any family, an error that occurred either because the family was mislabelled or because eggs from a non‐focal family in the hatchery were mistakenly planted out in the field. Individuals that were not fully assigned to a focal family were included in estimates of fry density and biomass but excluded from all other analyses (hence *n* = 29 families in some analyses).

### Statistical analyses

We tested the effects of parental nutrient levels on juvenile salmon and their macroinvertebrate prey using general linear and linear mixed models. Since residuals were not normally distributed for most dependent variables, we used a hierarchical bootstrapping approach to generate mean effects and *P*‐values in all analyses except those for selection gradients and differentials (Adèr & Adèr 2008). For analyses of dependent variables that were measured more than once in each stream – including macroinvertebrate abundance and biomass; fish length, density and biomass; and post‐selection means for egg mass, standard metabolic rate and maximum metabolic rate – we used linear mixed models with treatment (low vs. high parental nutrients) as a categorical fixed effect and stream as a random effect on the intercept. The bootstrap procedure for these analyses involved first sampling with replacement among values within each stream. Next, streams were sampled within treatments. The models were rerun 20 000 times, each time using a different sample of the data. Significance values were then calculated as a two‐tailed *P*‐value from the bootstrapped distribution of the treatment effect. Egg mass and maximum metabolic rate had an effect on survival (see Results) that might be explained by their effects on growth. To further tease apart these effects, we ran a model for growth that was similar to the above analyses except that we included egg mass, maximum metabolic rate and their interactions with treatment as fixed effects and family and stream as random effects.

To compare mean family diversity among stream types, we used a general linear model with treatment included as a fixed categorical effect. The bootstrap procedure for this analysis involved sampling, with replacement, values from each of the treatments and running the model with this resampled data. The model was rerun 20 000 times, each time using a different sample of the data. Significance of the effect of the nutrient level was then calculated as a two‐tailed *P*‐value from the bootstrapped distribution of the treatment effect. The number of fish captured per stream was included as a covariate in the analysis of family diversity, but was not statistically significant (*P *>* *0.05; presumably because similar numbers were collected in each stream) so was dropped from the model. For all analyses using the bootstrapping approach, 20 000 iterations were more than adequate to obtain convergence on the re‐sampled parameter estimates.

Survival, analysed as the number of surviving family members, followed a Poisson distribution, so selection differentials and gradients were analysed using generalised mixed models that specified a Poisson distribution and log link and included family and stream as random effects. None of the traits examined were correlated with one another at the family‐level (egg mass vs. standard metabolic rate: *r = *0.31, *P = *0.106; egg mass vs. maximum metabolic rate: *r = *0.05, *P = *0.806*;* standard metabolic rate vs. maximum metabolic rate: *r = *0.05, *P = *0.785).

Finally, we used the same linear mixed model approach as above to test whether the post‐selection variances in egg mass, standard metabolic rate, maximum metabolic rate and family level survival differed between streams with low vs. high nutrient levels. Log likelihood tests using the χ^2^ statistic were used to compare the fit of a model that included separate error variances for each treatment (low and high nutrients) vs. a reduced model that included a common variance. All tests were considered statistically significant when *P *<* *0.05.

## Results

**Figure 1:**
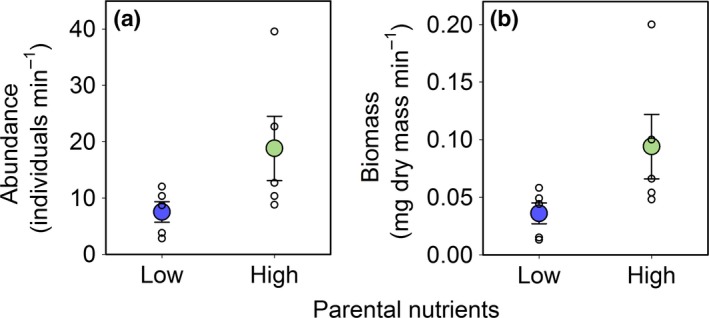
Macroinvertebrate prey of juvenile Atlantic salmon (*Salmo salar*) in streams with low (blue, *n* = 5) vs. high (green, *n* = 5) parental nutrient levels. Plotted are raw estimates of (a) total macroinvertebrate abundance and (b) total macroinvertebrate biomass for individual streams (unfilled circles) and the mean (filled circles; ± 1 SE) across streams within the low vs. high nutrient treatments. Estimates are given as the mean catch per unit effort for 1‐min samples taken at three locations at each of 50, 25 and 0 m above the downstream limit of each experimental reach.

**Figure 2:**
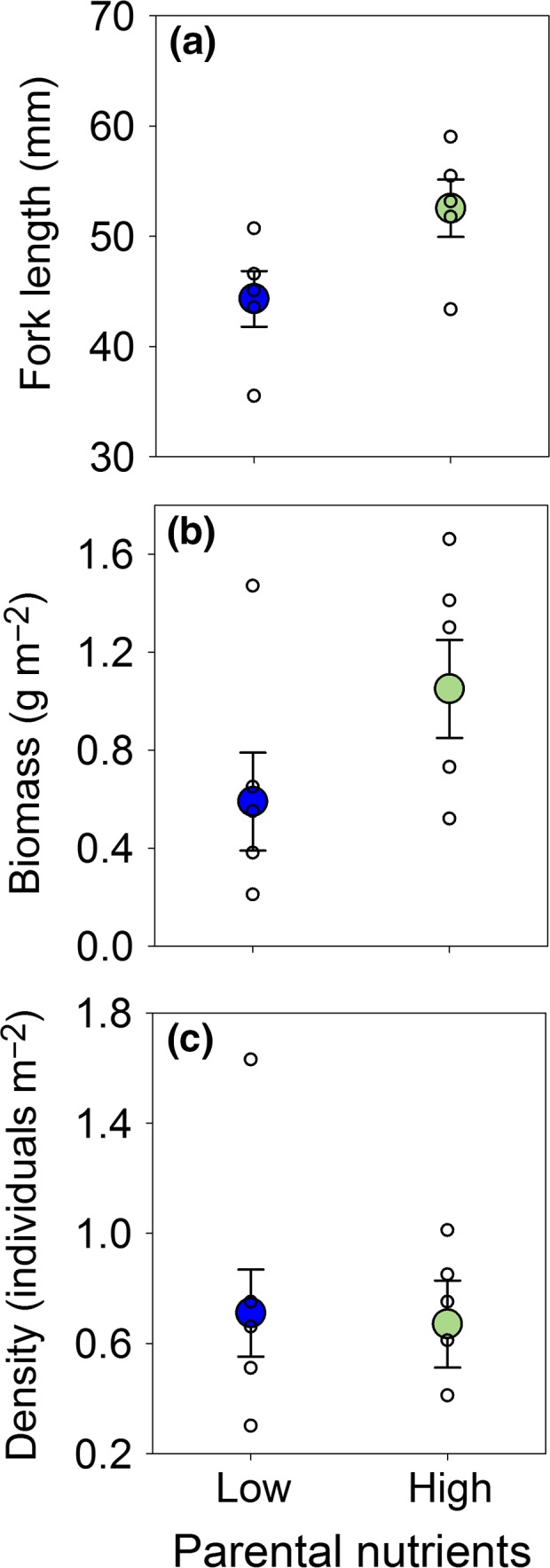
Growth, biomass and density of juvenile Atlantic salmon (*Salmo salar*) in streams with low (blue, *n* = 5) vs. high (green, *n* = 5) parental nutrient levels. Plotted are raw estimates for mean (± 1 SE) (a) fork length as a measure of growth, (b) biomass and (c) density for individual streams (unfilled circles) and the mean (filled circles; ± 1 SE) across streams within the low vs. high nutrient treatments. Fish density was estimated from depletion curves of the number of fish captured during triple‐pass electrofishing. Fish biomass was calculated as the product of the average fish body mass and the estimated density for each stream.

Nutrient levels had important effects on macroinvertebrate prey (Fig. 1). Macroinvertebrate populations in streams with low parental nutrients had roughly one‐third the abundance (*P *=* *0.042, Fig. 1a) and biomass (*P *=* *0.029, Fig. 1b) of streams with high parental nutrient levels, these differences in standing stocks being driven primarily by mayflies in the family Baetidae, a common prey item for juvenile salmon (Fig. S4). Macroinvertebrates were sampled at a critical time when juvenile salmon had only recently emerged from their gravel nests and begun to feed exogenously. As such, nutrient levels also influenced the somatic growth and body condition of the salmon. Specifically, juveniles in the low nutrient streams were smaller (*P *=* *0.001, Fig. 2a), resulting in a lower total fish biomass relative to streams with high nutrient levels (*P *=* *0.044, Fig. 2b) despite no differences in density among stream types (*P *=* *0.948, Fig. 2c).

**Figure 3:**
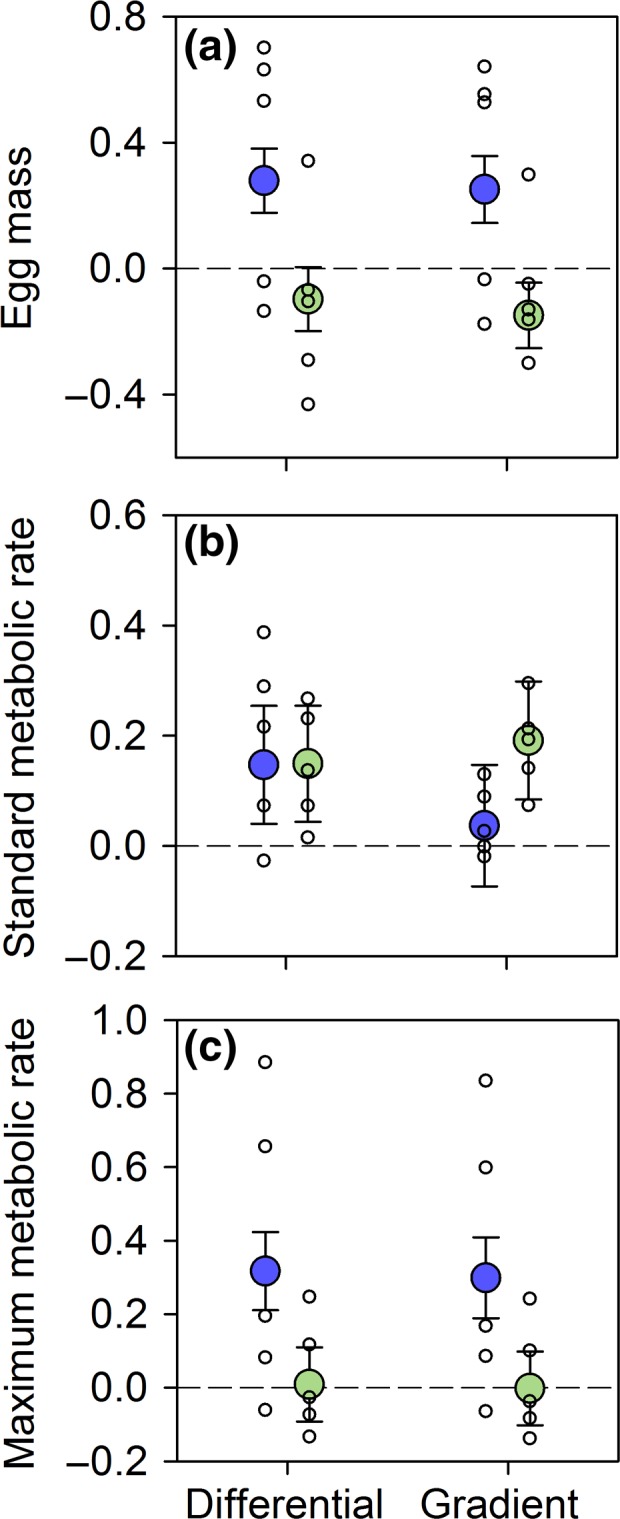
Selection differentials and gradients in streams with low (blue, *n* = 5) vs. high (green, *n* = 5) parental nutrient levels. Plotted are coefficients for standardised selection differentials and gradients for egg‐to‐juvenile survival (%) as a function of family level (a) egg mass, (b) standard metabolic rate and (c) maximum metabolic rate in full sibling Atlantic salmon (*Salmo salar*) families (*n* = 29) for individual streams (unfilled circles) and the mean (filled circles; ± 1 SE) across streams within the low vs. high nutrient treatments. See Table 1 for statistical analyses. Family level traits were not correlated with one another (see Methods), so selection differentials for all three traits showed qualitatively similar patterns to selection gradients.

Nutrient levels also affected the direction and magnitude of selection on two of the three energy‐related traits we examined. Specifically, there was strong directional selection for larger egg mass and higher maximum metabolic rate, but not standard metabolic rate, in streams with low parental nutrients (Table 1, Fig. 3, Fig. S5). In contrast, there was no detectable selection on egg mass, standard metabolic rate, or maximum metabolic rate in streams with high parental nutrients (Table 1, Fig. 2). Links between each of egg mass and maximum metabolic rate and survival could be mediated by their effects on growth. Indeed, egg mass had a positive effect on growth in streams with low nutrients (*P *=* *0.027) but no effect in streams with high nutrient levels (*P *=* *0.164). However, there was no effect of maximum metabolic rate on the growth of survivors in streams with either low (*P *=* *0.482) or high (*P *=* *0.459) nutrient levels. In addition, growth rate had no effect on the number of surviving family members in streams with either low (*P *=* *0.462) or high (*P *=* *0.221) parental nutrients.

**Table 1:** Standardised linear selection differentials and gradients (β ± 1 SE) for egg‐to‐juvenile survival (%) as a function of egg mass, standard metabolic rate (SMR) and maximum metabolic rate (MMR) of Atlantic salmon (*Salmo salar*) in streams with low vs. high levels of parental nutrients. Statistics are for tests of the difference of each differential and gradient (a) from zero and (b) between low and high nutrient streams. Differentials were calculated from generalised linear mixed models run separately for each trait while gradients were calculated from a generalised model including all traits as predictors of survival. Egg mass, SMR and MMR were not correlated with one another (see Methods), so selection differentials for all three traits showed qualitatively similar patterns to selection gradients. In addition, none of the quadratic selection gradients or differentials were statistically significant (see Table S2). Metabolic rates were standardised to a common body mass of 1 g prior to analyses

	Selection differential			Selection gradient		
	β ± 1 SE	*t*	*P*	β ± 1 SE	*t*	*P*
(a) Difference from zero						
Low nutrient streams						
Egg mass	0.28 ± 0.10	2.74	0.007	0.25 ± 0.10	2.36	0.019
SMR	0.15 ± 0.11	1.38	0.170	0.04 ± 0.11	0.33	0.740
MMR	0.32 ± 0.11	2.98	0.003	0.30 ± 0.11	2.83	0.005
High nutrient streams						
Egg mass	−0.10 ± 0.10	−0.96	0.338	−0.15 ± 0.10	−1.43	0.153
SMR	0.15 ± 0.11	1.41	0.158	0.19 ± 0.11	1.78	0.076
MMR	0.01 ± 0.10	0.09	0.929	−0.01 ± 0.10	−0.02	0.988
(b) Low vs. high nutrient streams						
Egg mass	0.38 ± 0.14	2.62	0.009	0.40 ± 0.15	2.69	0.008
SMR	0.00 ± 0.15	−0.01	0.991	−0.15 ± 0.15	−1.00	0.319
MMR	0.31 ± 0.15	2.03	0.037	0.30 ± 0.15	2.06	0.040

**Figure 4:**
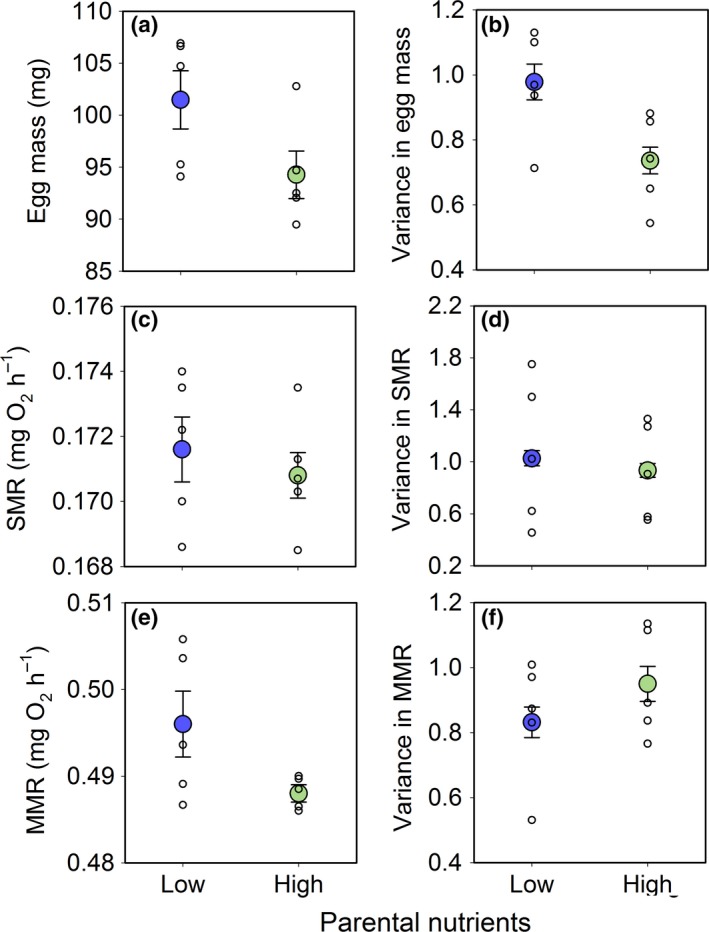
Phenotypic divergence in Atlantic salmon (*Salmo salar*) from streams with low (blue, *n* = 5) vs. high (green, *n* = 5) parental nutrients. Plotted are raw estimates for post‐selection means and variances of (a–b) egg mass, (c–d) standard metabolic rate (SMR) and (e–f) maximum metabolic rate (MMR) for individual streams (unfilled circles) and the mean (filled circles; ± 1 SE) across streams within the low vs. high nutrient treatments. Metabolic rates are standardised to a common body mass of 1 g. Eggs from 29 full sibling salmon families were planted out in equal density across the 10 streams, so any final differences among streams reflect the products of selection.

**Figure 5:**
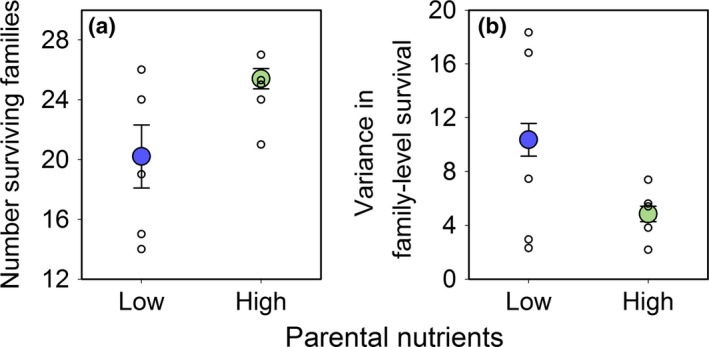
Genetic divergence in Atlantic salmon (*Salmo salar*) from streams with low (blue, *n* = 5) vs. high (green, *n* = 5) parental nutrients. Plotted are raw estimates for (a) the number of surviving families and (b) the variance in family level survival for individual streams (unfilled circles) and the mean (filled circles; ± 1 SE) across streams within the low vs. high nutrient treatments.

Differences in the intensity of selection among streams with low and high parental nutrient input led to rapid divergence at both the phenotypic and genetic level (Fig. 4 and 5). Notably, both the mean (*P *=* *0.015) and variance (χ^2^ = 12.5, *P *<* *0.001) in egg mass were significantly higher among surviving fish in streams with low compared to high nutrient levels (Fig. 4a,b). There was no difference in either the mean (*P *=* *0.535) or variance (χ^2^ = 1.2, *P *=* *0.137) of standard metabolic rate between stream types (Fig. 4c,d). In contrast, maximum metabolic rate had a higher mean (*P *=* *0.030) but lower variance (χ^2^ = 2.7, *P *=* *0.050) in streams with low compared to high nutrient levels (Fig. 4e,f). Finally, stronger directional selection led to a lower mean diversity of surviving families (*P *=* *0.048, Fig. 5a) but a higher variance in family level survival (χ^2^ = 20.35, *P *<* *0.001, Fig. 5b) in low compared to high nutrient streams.

## Discussion

Parental nutrient levels had important ecological effects and evolutionary consequences for offspring. Increases in prey availability, juvenile growth and juvenile biomass are consistent with previous studies of Atlantic salmon populations (Williams *et al*. 2009; Guyette *et al*. 2013) and other migratory fish species (Naiman *et al*. 2002; Childress *et al*. 2014). However, the evolutionary consequences of parental nutrient input are less well understood. Our results demonstrate that parental nutrients can alter selection pressures on key energy‐related traits with important consequences for phenotypic and genetic diversity. Below we discuss the implications of these results for our understanding of the evolution of these traits and their potential role in the eco‐evolutionary dynamics of wild populations.

Differences in selection intensity among streams that vary in parental nutrient input provide key insights into how energy‐related traits such as egg size and metabolic rate evolve in response to environmental productivity. Competition will be most severe when resources are limiting, so increased selection for larger egg size under poor growth conditions is predicted on theoretical grounds (Parker & Begon 1986; Einum *et al*. 2004; Rollinson & Hutchings 2013b). However, to date this has been demonstrated empirically primarily through short‐term food manipulation experiments using confined or laboratory populations (Hutchings 1991; Einum & Fleming 1999; Einum *et al*. 2004; Dziminski & Roberts 2006; Krist 2011). There is some correlative evidence that the early growth and survival advantages of a larger egg size can depend on different metrics of environmental quality in the wild (Krist 2011; Rollinson & Hutchings 2013a). Our manipulation of nutrient levels and prey availability over multiple streams and over timescales of months provides direct experimental evidence for these expected patterns of selection in wild populations. In addition, we found that families with larger initial egg masses grew faster in low nutrient streams, suggesting that egg mass may have indirect effects on survival via growth rate. However, faster growth and larger body size are not always beneficial for survival (Dibattista *et al*. 2007; Carlson *et al*. 2008). There was no evidence for an effect of growth on survival in our study. Our experimental design limited our analyses to surviving families only though, so further research is needed to tease apart the links between egg mass, growth and survival.

Selection for higher maximum metabolic rates observed in streams with low parental nutrients contrasts with results from laboratory studies showing that a lower metabolic rate is advantageous in poor quality environments (Bochdansky *et al*. 2005; Burton *et al*. 2011; Auer *et al*. 2015). Instead, our experimental evaluation of selection in the wild supports the alternative hypothesis that a high metabolic rate is favoured when competition is more intense. Individuals with higher metabolic rates are more likely to be dominant (Metcalfe *et al*. 1995; Biro & Stamps 2010) and thereby have priority access to more favourable areas, i.e. those with high food availability and low predation risk (Metcalfe *et al*. 2016). This may be critical to early survival, particularly in territorial species, since individuals that fail to establish feeding or refuge sites tend to be displaced and suffer higher mortality (Stamps 1983; Elliott 1986, 1990). Laboratory studies demonstrate that metabolic rate can have either positive or negative effects on growth rate depending on food supply (Burton *et al*. 2011). There was no evidence in this study for an effect of metabolic rate on growth rate, nor of growth on survival in the wild. This suggests that the observed impact of metabolic rate on survival was mediated through its effect on the ability to acquire territories rather than any direct effect on growth rates.

Strong selection for larger egg mass and higher maximum metabolic rate in streams with low parental nutrients suggests the possibility for a relatively rapid evolutionary response, even under the moderate levels of heritability reported for egg mass in salmon (narrow‐sense heritability *h* ^2^ ≥ 0.50; Kinnison *et al*. 2001) and maximum metabolic rate in vertebrates (mean ± 1 SE *h* ^2^ = 0.49 ± 0.07 calculated from White & Kearney 2013). Indeed, egg mass and maximum metabolic rate diverged rapidly (within months) between stream types. While our analysis is limited to the egg‐to‐juvenile stage of life, patterns of selection during this critical early stage are likely to play an important role in shaping longer term evolutionary changes. In age‐structured populations, population growth rate and individual fitness respond more or less strongly to changes in natural selection at different ages (Hamilton 1966; Charlesworth 1994). Small changes in demographic rates (e.g. survival) of younger age classes typically have larger effects on fitness than do equivalent changes in demographic rates of older age classes. This is especially true in organisms where mortality during the egg‐to‐juvenile stage is often greater than 90%, as found here (Bradford 1995; Klemetsen *et al*. 2003; Bassar *et al*. 2016). As such, patterns of selection observed here are likely to have far reaching implications for fitness differences among families and overall growth (mean fitness) of these populations.

Observed effects of parental nutrients on patterns of family level survival provide experimental evidence for key components of eco‐evolutionary feedbacks in wild populations: organisms can modify their surroundings to a large enough extent that these changes then alter their own selective environment (Post & Palkovacs 2009; Matthews *et al*. 2014; Hendry 2017). Alteration of selection pressures on egg size and metabolic rates may, in turn, feed back to influence future numbers of spawning parents and their nutrient subsidies, thereby leading to reciprocal interactions between ecological and evolutionary processes. However, the ecological consequences of evolutionary changes in these traits are likely to be complex. For example selection for increased egg size invariably comes at a cost to egg production (Einum *et al*. 2004); reduced egg production, in turn, may reduce the density of spawning adults but also lead indirectly to increased nutrient export by emigrating juveniles (Nislow *et al*. 2004; Moore *et al*. 2011), thereby creating a negative feedback that leads to further selection for larger egg size. In contrast, selection for increased aerobic capacity could have a positive impact on the number of spawning parents by enhancing their survival during migration (Martin *et al*. 2015; Eliason & Farrell 2016).

Nutrient subsidies and their effects on ecological and evolutionary processes are also likely to vary both spatially and temporally. Nutrient input depends on population size and mortality rates on the spawning grounds (Wipfli *et al*. 1999; Jonsson & Jonsson 2003; Flecker *et al*. 2010). Incorporation of nutrients into the freshwater and surrounding terrestrial environment, in turn, depends on factors such as temperature, light levels and stream discharge (Naiman *et al*. 2002; Gende *et al*. 2004). Furthermore, nutrient subsidies are decreasing in some freshwater lakes and rivers due in part to population declines of anadromous fish species but also to other factors such as barriers to migration, active nutrient removal and the net export of nutrients by seaward migrating fish stocked in streams above dams that impede returning adults (Parrish *et al*. 1998; Gresh *et al*. 2000; Stockner *et al*. 2000; Nislow *et al*. 2004; Flecker *et al*. 2010; Moore *et al*. 2011). In other systems, however, nutrient subsidies from historically abundant anadromous fish have been replaced by anthropogenic inputs (e.g. via wastewater or agricultural runoff; Twining *et al*. 2013) or by introduced species (Twining *et al*. 2016). Selection pressures may therefore vary over time and among systems with different background nutrient levels such that small changes in nutrient subsidies have large impacts on selection in ultra‐oligotrophic systems but little or no impact in more productive systems.

Our study is limited to one episode of selection, so it is not yet clear if observed selection pressures cause evolutionary changes over the long‐term or if they lead to eco‐evolutionary feedbacks between parental nutrient levels and organismal traits. Nevertheless, there is increasing evidence that nutrient subsidies from migratory fishes provide multiple opportunities for interactions between ecological and evolutionary processes at both the population and community level (Flecker *et al*. 2010; Hendry 2017). For example salmon carcasses can influence the genotypic effects of riparian tree species on ecosystem parameters (LeRoy *et al*. 2015). There is also evidence that selection on salmon migrating to their spawning grounds can alter nutrient dynamics; selection against larger sized Pacific salmon due to stranding and bear predation leads to changes in their size distribution and a subsequent decrease and increase, respectively, in the flux of nutrients into the spawning grounds (Carlson *et al*. 2011; Hendry 2017). Our study shows that changes in these nutrient subsidies can alter selection pressures on the fish themselves and influence subsequent trait distributions, thereby providing additional evidence for the importance of migratory fishes as mediators of eco‐evolutionary dynamics in the wild.

## Author Contributions

SKA, GJA, SM, DM, RDB, JDA, KHN and NBM contributed to the conceptual development and experimental planning of the study. SKA, GJA, SM, DM and HKD undertook the artificial fertilisation of salmon eggs. SKA, GJA and SM planted eggs and analogue carcasses out into streams. SM and LM reared salmon eggs in the hatchery and sampled invertebrates. SKA, GJA and DM measured egg mass and juvenile metabolic rates. SKA, GJA, SM, RDB, HKD, LM, TAJM, KS, DO, AG and NBM recaptured and measured fish. HKD and TAJM measured stream habitat parameters. GJA and TR sorted, processed and measured invertebrates with input from SKA and RDB. Data were analysed by SKA and RDB with input from NBM. The manuscript was written by SKA with input and final approval from all authors.
