# Supplementary material for: Nutrients from salmon parents alter selection pressures on their offspring
Source: Ecol Lett. 2017 Dec 15;21(2):287–95. doi: 10.1111/ele.12894 (PMC5814727; doi:10.1111/ele.12894)
Supplement: Supplementary file 1 [file ELE-21-287-s001.pdf]

SUPPLEMENTARY INFORMATION

Type of paper: Letter

Title: Nutrients from salmon parents alter selection pressures on their offspring

Authors and affiliations: Sonya K. Auer[1*], Graeme J. Anderson[1], Simon McKelvey[2], Ronald D. Bassar[3], Darryl McLennan[1], John D. Armstrong[4], Keith H. Nislow[5], Helen K. Downie[4], Lynn McKelvey[2], Thomas A.J. Morgan[4], Karine Salin[1], Danielle L. Orrell[1], Alice Gauthey[6], Thomas C. Reid[1], and Neil B. Metcalfe[1]

[1] Institute of Biodiversity, Animal Health and Comparative Medicine, University of Glasgow, Glasgow, United Kingdom.

[2] Cromarty Firth Fishery Trust, Inverness, United Kingdom.

[3] Department of Biology, Williams College, Massachusetts, USA.

[4] Marine Scotland – Science, Freshwater Fisheries Laboratory, Pitlochry, United Kingdom.

[5] USDA Forest Service Northern Research Station, Amherst, MA USA.

[6] Université Pierre et Marie Curie, Paris, France.

*Correspondence to: sonya.auer@gmail.com

Table S1. Location, sampling times, and characteristics of ten study streams in the northern highlands of Scotland. Nutrients refers to whether streams had low or high parental nutrient levels; the latter each received carcass-analogs in an amount equivalent to 25 salmon carcasses. Parr densities were estimated as number of parr captured divided by the area sampled.

**Stream**	**Location (Lat./Long.)**	**Nutrients**	**Egg and carcass planting**	**Invertebrate sampling**	**Fish capture**	**Mean stream wet width (m)**	**Mean parr density (per m^2)**
A Chomair	57.595 / -5.002	Low	07/03/16	31/05/16	15/7/16	6.95	0.042
An Eilean Ghuirm	57.706 / -4.696	Low	25/02/16	31/05/16	11/7/16	2.54	0.024
Chaiseachain	57.598 / -4.949	High	25/02/16	08/06/16	23/7/16	2.93	0.025
Coire a Ghormachain	57.549 / -5.129	High	03/03/16	04/06/16	12/7/16	4.50	0.017
Coire nan Laogh	57.581 / -5.059	Low	03/03/16	04/06/16	16/7/16	4.54	0.046
Coire a Bhuic	57.513 / -5.001	Low	26/02/16	02/06/16	13/7/16	4.56	0.000
Gleann Chorain	57.500 / -4.916	High	26/02/16	07/06/16	21/7/16	5.03	0.008
Mhartuin	57.555 / -5.096	Low	03/03/16	04/06/16	24/7/16	6.57	0.020
Scardroy	57.519 / -4.992	High	26/02/16	02/06/16	22/7/16	6.40	0.012
Gleann Meinich	57.543 / -4.935	High	26/02/16	10/06/16	14/7/16	6.39	0.021

Table S2. Standardised quadratic selection differentials and gradients (β ±1SE) for egg-to-juvenile survival (%) as a function of egg mass, mass-independent standard metabolic rate (SMR), and mass-independent maximum metabolic rate (MMR) of Atlantic salmon (*Salmo salar*) in streams with low versus high levels of parental nutrients. Statistics are for tests of the difference of each differential and gradient a) from zero and b) between low and high nutrient streams. Differentials were calculated from generalized linear mixed models run separately for each trait while gradients were calculated from a generalized model including all traits as predictors of survival. Egg mass, SMR, and MMR were not correlated with one another (see methods), so selection differentials for all three traits showed qualitatively similar patterns to selection gradients.

	**Selection differential**			**Selection gradient**		
	β ± 1SE	*t*	*P*	β ± 1SE	*t*	*P*
**a) Difference from zero**						
**Low nutrient streams**						
Egg mass	0.23 ± 0.11	2.06	0.041	0.24 ± 0.11	2.06	0.040
Egg mass^2	0.11 ± 0.12	0.91	0.364	0.08 ± 0.12	0.63	0.531
SMR	0.11 ± 0.12	0.87	0.385	-0.03 ± 0.13	-0.19	0.848
SMR^2	-0.04 ± 0.07	-0.62	0.537	-0.08 ± 0.07	-1.12	0.265
MMR	0.31 ± 0.11	2.89	0.004	0.29 ± 0.11	2.61	0.010
MMR^2	-0.03 ± 0.08	-0.41	0.686	-0.09 ± 0.09	-1.03	0.302
**High nutrient streams**						
Egg mass	-0.04 ± 0.11	-0.34	0.735	-0.09 ± 0.11	-0.83	0.407
Egg mass^2	-0.17 ± 0.12	-1.44	0.151	-0.14 ± 0.12	-1.15	0.252
SMR	0.04 ± 0.12	0.36	0.722	0.16 ± 0.13	1.23	0.221
SMR^2	-0.11 ± 0.08	-1.36	0.176	-0.12 ± 0.07	-1.57	0.117
MMR	-0.04 ± 0.11	-0.40	0.688	-0.01 ± 0.11	-0.12	0.907
MMR^2	-0.14 ± 0.08	-1.84	0.067	-0.14 ± 0.12	-1.83	0.068
**b) Difference between low and high nutrient streams**						
Egg mass	0.27 ± 0.16	1.71	0.088	0.33 ± 0.16	2.06	0.040
Egg mass^2	0.27 ± 0.16	1.66	0.098	0.21 ± 0.17	1.25	0.211
SMR	0.07 ± 0.17	0.37	0.708	-0.18 ± 0.18	-0.99	0.322
SMR^2	0.09 ± 0.11	0.88	0.379	0.04 ± 0.10	0.34	0.734
MMR	0.35 ± 0.15	2.33	0.020	0.30 ± 0.16	1.93	0.054
MMR^2	0.11 ± 0.11	0.97	0.332	0.06 ± 0.11	0.48	0.635

Table S3. Published coefficients for length-mass equations (DM = aL^b where DM = dry mass in mg and L = length in mm) for aquatic larvae from macroinvertebrate families in two different taxonomic orders. Equations are from Benke, A.C., et al. (1999) Length-mass relationships from freshwater macroinvertebrates in North America with particular reference to the southeastern United States. Journal of the North American Benthological Society, 18, 308-343.

**Order**	**Family**	***a***	***b***
**Ephemeroptera**	Baetidae	0.0053	2.875
	Ephemerellidae	0.0103	2.676
**Diptera**	Chironomidae	0.0018	2.617
	Simuliidae	0.0020	3.011

Figure S1. Map of River Conon catchment in Northern Scotland, including the location of study streams (blue = low and green = high parental nutrient levels), key hydroelectric dams, and trap for collecting returning adult salmon on their spawning migration.

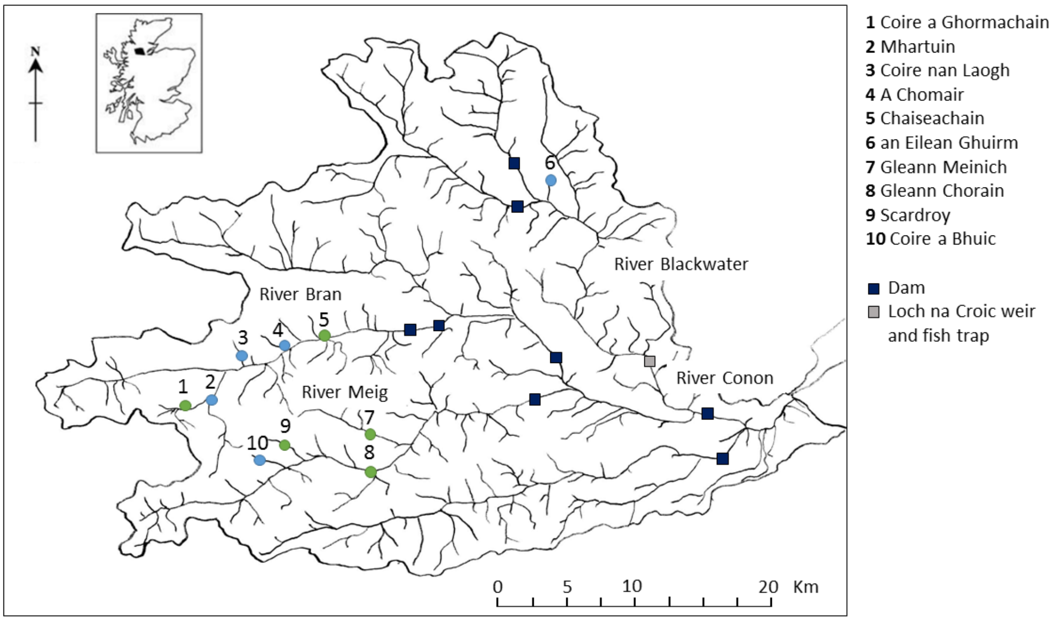

Figure S2. Mean (±1SE) standard (white) and maximum (black) metabolic rate of 29 full-sibling Atlantic salmon (*Salmo salar*) families measured at 12˚C. Metabolic rates are standardised to a common body mass of 1g.

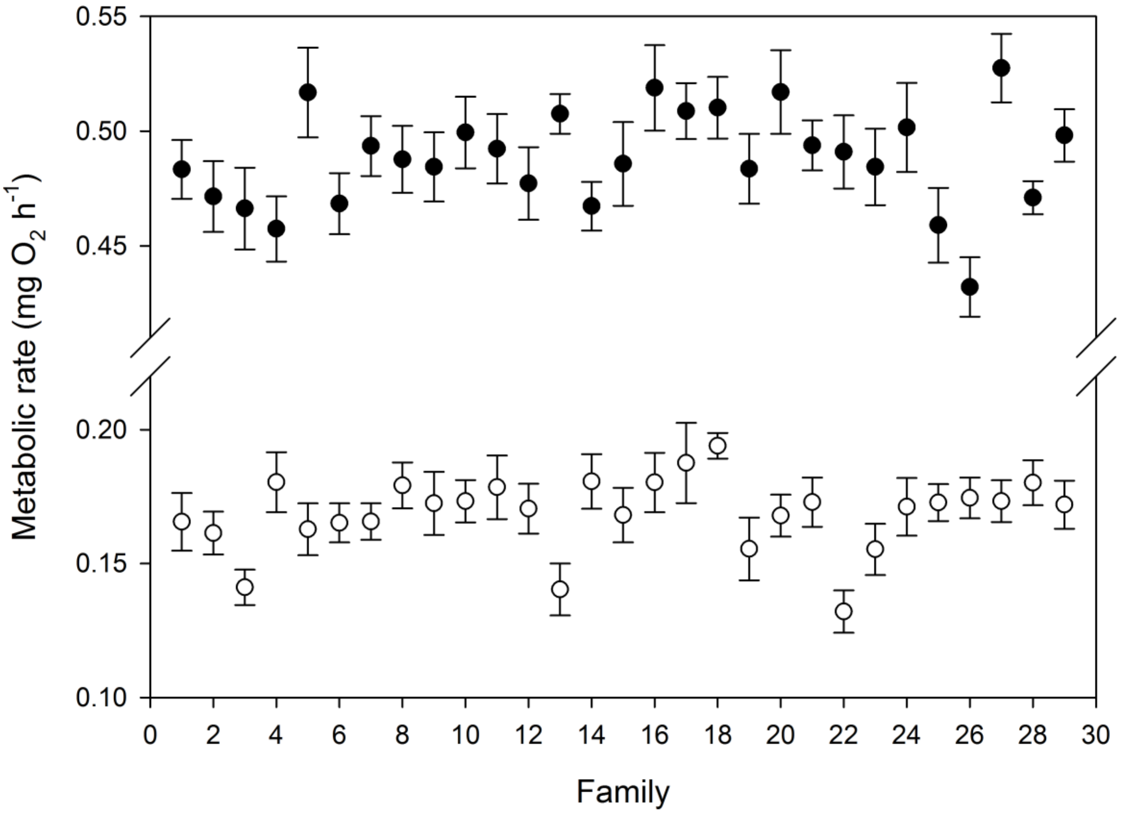

Figure S3. Temporal changes in mean (±1SE) daily water temperature in eight study streams from the time eggs were planted out to their recapture as juveniles. Temperature loggers were placed out in all ten study streams, but two of them malfunctioned.

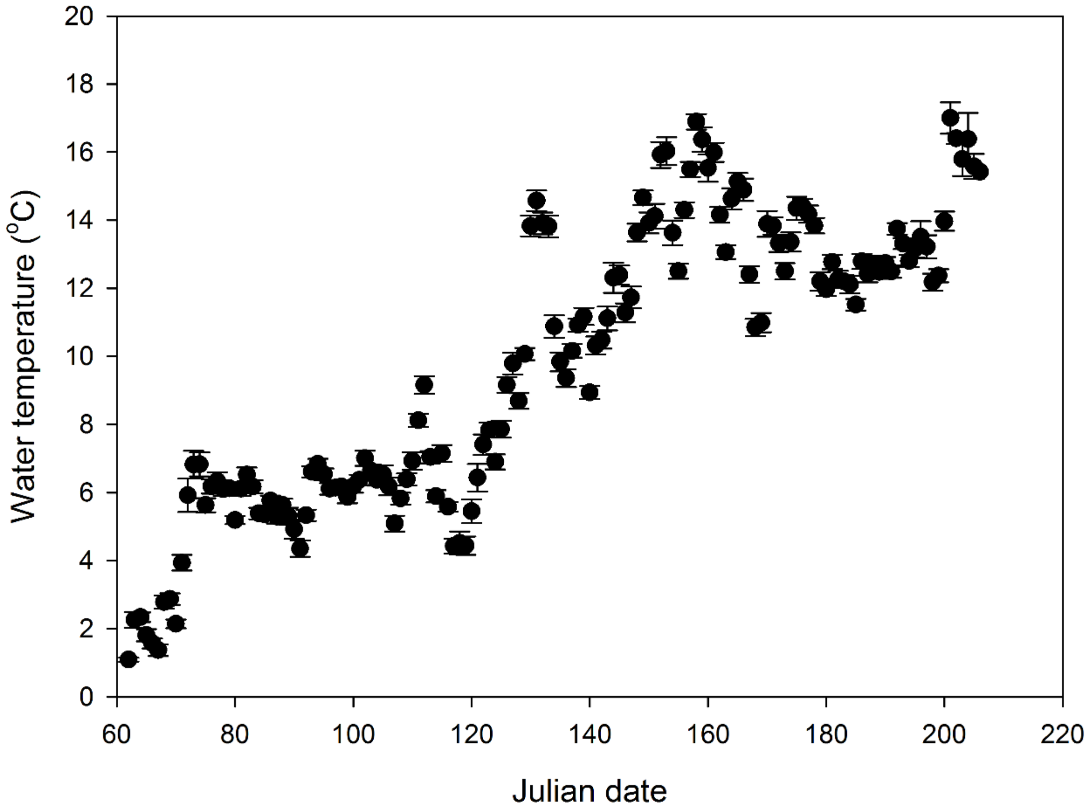

Figure S4. Macroinvertebrate prey of juvenile Atlantic salmon (*Salmo salar*) in streams with low (blue, n = 5) versus high (green, n = 5) parental nutrient levels. Plotted are raw estimates of mean (± 1SE) abundance and biomass for each macroinvertebrate family. Estimates are given as the mean catch of prey equal to or less than 1mm in width (maximum prey size of salmon fry) per unit effort (1 min electrobugging), with samples taken at three locations at each of 50, 25, and 0 m above the downstream limit of each experimental reach.

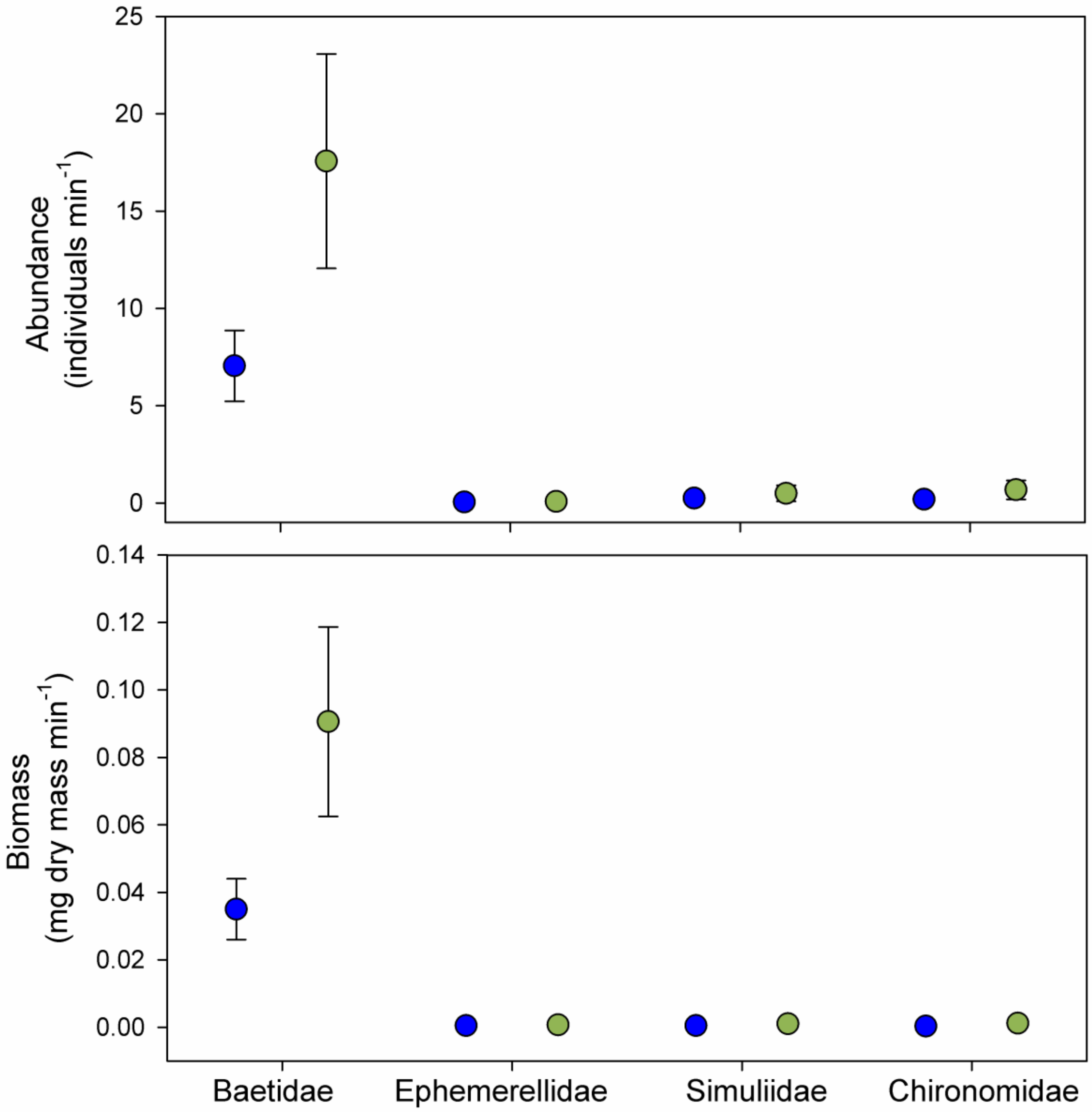

Figure S5. Linear selection gradients in streams with low (blue, n = 5) versus high (green, n = 5) parental nutrients. Plotted are standardized selection gradients for egg-to-juvenile survival (%) as a function of (a) egg mass, (b) standard metabolic rate, and (c) maximum metabolic rate in full sibling Atlantic salmon (*Salmo salar*) families (n = 29). Metabolic rates were standardised to a common body mass of 1g prior to analyses. See Table 1 and S2 for parameter estimates and statistical details.

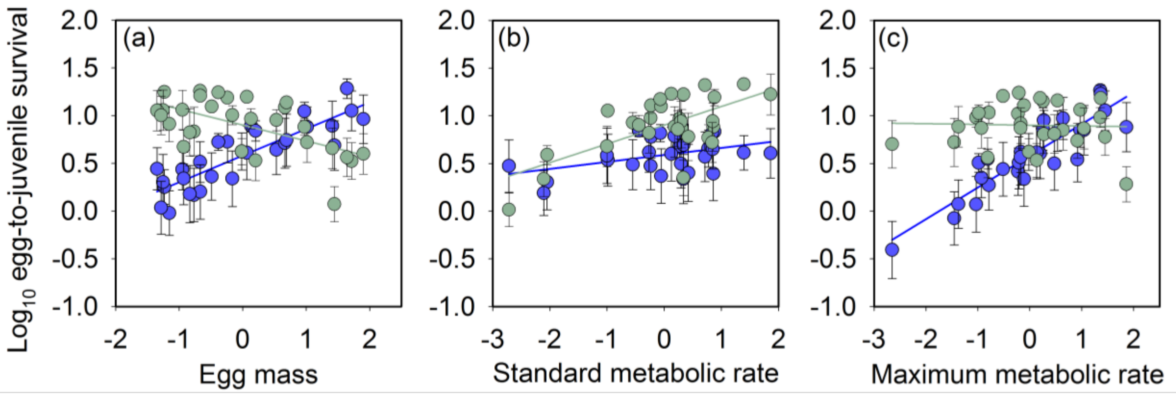

SUPPLEMENTARY DATA

1) INVERTEBRATE ABUNDANCE AND BIOMASS

stream	treatment	section	abundance (CPUE)	biomass (CPUE)
1	Low	50	6	0.032
1	Low	75	14.333	0.068
1	Low	100	15.667	0.075
2	Low	50	2	0.008
2	Low	75	4.667	0.02
2	Low	100	4.667	0.012
3	High	50	9.333	0.049
3	High	75	16.667	0.088
3	High	100	12	0.061
4	High	50	55.333	0.286
4	High	75	20	0.1
4	High	100	43.333	0.215
5	Low	50	5.667	0.029
5	Low	75	9.333	0.045
5	Low	100	11	0.058
6	Low	50	15.333	0.069
6	Low	75	14.667	0.074
6	Low	100	1	0.005
7	High	50	17.667	0.092
7	High	75	6.333	0.034
7	High	100	7	0.036
8	Low	50	5	0.028
8	Low	75	0.667	0.004
8	Low	100	2.667	0.013
9	High	50	11.333	0.063
9	High	75	12.667	0.067
9	High	100	2.333	0.014
10	High	50	12.667	0.055
10	High	75	28.667	0.118
10	High	100	26.667	0.126

2) FISH DENSITY AND BIOMASS

stream	Treatment	Density (m^2)	Biomass (m^2)
1	Low	0.66	0.65
2	Low	0.51	0.21
3	High	0.75	1.66
4	High	0.61	0.52
5	Low	1.63	1.47
6	Low	0.75	0.55
7	High	1.01	1.41
8	Low	0.3	0.38
9	High	0.41	0.73
10	High	0.85	1.3

3) TRAITS OF SURVIVORS

family	stream	treatment	length (mm)	eggmass (mg)	SMR (mg O_2 h^{-1})	MMR (mg O_2 h^{-1})	Jdate	density (per m^2)
24	1	Low	39.09	74.8	0.171	0.502	197	0.657
21	1	Low	51.28	121.06	0.173	0.494	197	0.657
16	1	Low	42.16	122.22	0.18	0.519	197	0.657
29	1	Low	50.28	98.82	0.18	0.471	197	0.657
16	1	Low	46.4	122.22	0.18	0.519	197	0.657
28	1	Low	53.17	118	0.166	0.527	197	0.657
5	1	Low	40.55	97.7	0.163	0.517	197	0.657
3	1	Low	40.01	93.13	0.146	0.466	197	0.657
16	1	Low	47.74	122.22	0.18	0.519	197	0.657
3	1	Low	43.43	93.13	0.146	0.466	197	0.657
24	1	Low	37.31	74.8	0.171	0.502	197	0.657
16	1	Low	47.06	122.22	0.18	0.519	197	0.657
16	1	Low	47.47	122.22	0.18	0.519	197	0.657
29	1	Low	43.76	98.82	0.18	0.471	197	0.657
16	1	Low	47.16	122.22	0.18	0.519	197	0.657
24	1	Low	37.91	74.8	0.171	0.502	197	0.657
24	1	Low	37.34	74.8	0.171	0.502	197	0.657
7	1	Low	45.28	91.94	0.166	0.493	197	0.657
16	1	Low	47.18	122.22	0.18	0.519	197	0.657
28	1	Low	50.56	118	0.166	0.527	197	0.657
5	1	Low	44.27	97.7	0.163	0.517	197	0.657
29	1	Low	53.57	98.82	0.18	0.471	197	0.657
28	1	Low	51.97	118	0.166	0.527	197	0.657
16	1	Low	46.96	122.22	0.18	0.519	197	0.657
28	1	Low	51.85	118	0.166	0.527	197	0.657

16	1	Low	45.79	122.22	0.18	0.519	197	0.657
16	1	Low	46.48	122.22	0.18	0.519	197	0.657
20	1	Low	48.18	89.72	0.177	0.517	197	0.657
16	1	Low	48.76	122.22	0.18	0.519	197	0.657
21	1	Low	48.68	121.06	0.173	0.494	197	0.657
29	1	Low	43.24	98.82	0.18	0.471	197	0.657
16	1	Low	48.88	122.22	0.18	0.519	197	0.657
29	1	Low	43.22	98.82	0.18	0.471	197	0.657
21	1	Low	48.78	121.06	0.173	0.494	197	0.657
5	1	Low	47.24	97.7	0.163	0.517	197	0.657
12	1	Low	48.54	96.67	0.17	0.477	197	0.657
21	1	Low	48.47	121.06	0.173	0.494	197	0.657
5	1	Low	48.62	97.7	0.163	0.517	197	0.657
16	1	Low	43.8	122.22	0.18	0.519	197	0.657
24	1	Low	34.97	74.8	0.171	0.502	197	0.657
16	1	Low	47.75	122.22	0.18	0.519	197	0.657
16	1	Low	46.9	122.22	0.18	0.519	197	0.657
24	1	Low	44.27	74.8	0.171	0.502	197	0.657
21	1	Low	48.81	121.06	0.173	0.494	197	0.657
28	1	Low	52.77	118	0.166	0.527	197	0.657
7	1	Low	42.69	91.94	0.166	0.493	197	0.657
16	1	Low	49.95	122.22	0.18	0.519	197	0.657
12	1	Low	41.04	96.67	0.17	0.477	197	0.657
16	1	Low	50.61	122.22	0.18	0.519	197	0.657
16	1	Low	48.87	122.22	0.18	0.519	197	0.657
17	1	Low	48.27	76.56	0.188	0.509	197	0.657
29	1	Low	43.8	98.82	0.18	0.471	197	0.657
16	1	Low	48.83	122.22	0.18	0.519	197	0.657
16	1	Low	47.47	122.22	0.18	0.519	197	0.657
16	1	Low	50.89	122.22	0.18	0.519	197	0.657

12	1	Low	49.16	96.67	0.17	0.477	197	0.657
7	1	Low	43.37	91.94	0.166	0.493	197	0.657
28	1	Low	48.29	118	0.166	0.527	197	0.657
12	1	Low	43.4	96.67	0.17	0.477	197	0.657
16	1	Low	47.4	122.22	0.18	0.519	197	0.657
5	1	Low	44.66	97.7	0.163	0.517	197	0.657
16	1	Low	47.29	122.22	0.18	0.519	197	0.657
16	1	Low	48.48	122.22	0.18	0.519	197	0.657
28	1	Low	50.34	118	0.166	0.527	197	0.657
28	1	Low	56.41	118	0.166	0.527	197	0.657
24	1	Low	37.86	74.8	0.171	0.502	197	0.657
29	1	Low	48.02	98.82	0.18	0.471	197	0.657
12	1	Low	41	96.67	0.17	0.477	197	0.657
29	1	Low	47.4	98.82	0.18	0.471	197	0.657
16	1	Low	48.91	122.22	0.18	0.519	197	0.657
5	1	Low	41.16	97.7	0.163	0.517	197	0.657
5	1	Low	47.8	97.7	0.163	0.517	197	0.657
16	1	Low	46	122.22	0.18	0.519	197	0.657
28	1	Low	49.33	118	0.166	0.527	197	0.657
29	1	Low	50.2	98.82	0.18	0.471	197	0.657
16	1	Low	47.9	122.22	0.18	0.519	197	0.657
16	1	Low	50.26	122.22	0.18	0.519	197	0.657
21	1	Low	47.68	121.06	0.173	0.494	197	0.657
16	1	Low	48.45	122.22	0.18	0.519	197	0.657
16	1	Low	47.84	122.22	0.18	0.519	197	0.657
28	1	Low	50.48	118	0.166	0.527	197	0.657
16	1	Low	53.44	122.22	0.18	0.519	197	0.657
21	1	Low	48.54	121.06	0.173	0.494	197	0.657
24	1	Low	42.98	74.8	0.171	0.502	197	0.657
24	1	Low	37.97	74.8	0.171	0.502	197	0.657

24	1	Low	40.85	74.8	0.171	0.502	197	0.657
21	1	Low	50.99	121.06	0.173	0.494	197	0.657
29	1	Low	44.26	98.82	0.18	0.471	197	0.657
5	1	Low	43.45	97.7	0.163	0.517	197	0.657
3	1	Low	45.25	93.13	0.146	0.466	197	0.657
24	1	Low	40.6	74.8	0.171	0.502	197	0.657
5	1	Low	40.84	97.7	0.163	0.517	197	0.657
28	1	Low	53.96	118	0.166	0.527	197	0.657
16	1	Low	50.21	122.22	0.18	0.519	197	0.657
14	1	Low	52.58	110.71	0.181	0.467	197	0.657
29	1	Low	44.05	98.82	0.18	0.471	197	0.657
16	1	Low	42.01	122.22	0.18	0.519	197	0.657
24	1	Low	38.81	74.8	0.171	0.502	197	0.657
24	1	Low	40.84	74.8	0.171	0.502	197	0.657
6	1	Low	48.37	111.35	0.165	0.468	197	0.657
28	1	Low	49.44	118	0.166	0.527	197	0.657
16	1	Low	47.86	122.22	0.18	0.519	197	0.657
29	1	Low	51.07	98.82	0.18	0.471	197	0.657
16	1	Low	49.56	122.22	0.18	0.519	197	0.657
20	1	Low	48.63	89.72	0.177	0.517	197	0.657
16	1	Low	48.11	122.22	0.18	0.519	197	0.657
16	1	Low	50.58	122.22	0.18	0.519	197	0.657
24	1	Low	42.21	74.8	0.171	0.502	197	0.657
24	1	Low	39.19	74.8	0.171	0.502	197	0.657
10	1	Low	44.92	85.28	0.172	0.499	197	0.657
5	1	Low	47.79	97.7	0.163	0.517	197	0.657
16	1	Low	49.17	122.22	0.18	0.519	197	0.657
16	1	Low	48.18	122.22	0.18	0.519	197	0.657
5	1	Low	44.48	97.7	0.163	0.517	197	0.657
5	1	Low	52.57	97.7	0.163	0.517	197	0.657

16	1	Low	49.9	122.22	0.18	0.519	197	0.657
24	1	Low	38.38	74.8	0.171	0.502	197	0.657
28	1	Low	50.73	118	0.166	0.527	197	0.657
5	1	Low	39.47	97.7	0.163	0.517	197	0.657
21	1	Low	48.72	121.06	0.173	0.494	197	0.657
24	1	Low	46.94	74.8	0.171	0.502	197	0.657
8	1	Low	47.44	95.41	0.179	0.488	197	0.657
28	1	Low	46.49	118	0.166	0.527	197	0.657
16	1	Low	54.17	122.22	0.18	0.519	197	0.657
16	1	Low	46.03	122.22	0.18	0.519	197	0.657
16	1	Low	51.31	122.22	0.18	0.519	197	0.657
29	1	Low	41.53	98.82	0.18	0.471	197	0.657
2	2	Low	33.5	103.88	0.161	0.471	193	0.509
18	2	Low	39.97	117.5	0.192	0.51	193	0.509
20	2	Low	33.09	89.72	0.177	0.517	193	0.509
18	2	Low	34.19	117.5	0.192	0.51	193	0.509
8	2	Low	33.88	95.41	0.179	0.488	193	0.509
2	2	Low	33.81	103.88	0.161	0.471	193	0.509
11	2	Low	35.16	105.96	0.178	0.492	193	0.509
21	2	Low	40.95	121.06	0.173	0.494	193	0.509
22	2	Low	32.27	81.3	0.132	0.491	193	0.509
23	2	Low	29.97	76.25	0.155	0.484	193	0.509
21	2	Low	38.83	121.06	0.173	0.494	193	0.509
23	2	Low	30.72	76.25	0.155	0.484	193	0.509
19	2	Low	38.27	106.35	0.148	0.484	193	0.509
23	2	Low	28.89	76.25	0.155	0.484	193	0.509
9	2	Low	30.13	80.99	0.172	0.484	193	0.509
21	2	Low	34.68	121.06	0.173	0.494	193	0.509
1	2	Low	33.15	88.13	0.173	0.483	193	0.509
18	2	Low	38.65	117.5	0.192	0.51	193	0.509

18	2	Low	36.59	117.5	0.192	0.51	193	0.509
30	2	Low	29.7	75.77	0.172	0.498	193	0.509
2	2	Low	31.36	103.88	0.161	0.471	193	0.509
23	2	Low	37.34	76.25	0.155	0.484	193	0.509
18	2	Low	41.62	117.5	0.192	0.51	193	0.509
30	2	Low	32.2	75.77	0.172	0.498	193	0.509
18	2	Low	33.25	117.5	0.192	0.51	193	0.509
16	2	Low	34.02	122.22	0.18	0.519	193	0.509
16	2	Low	30.28	122.22	0.18	0.519	193	0.509
18	2	Low	35.26	117.5	0.192	0.51	193	0.509
19	2	Low	39.52	106.35	0.148	0.484	193	0.509
18	2	Low	35.09	117.5	0.192	0.51	193	0.509
26	2	Low	36.99	125.1	0.173	0.459	193	0.509
22	2	Low	37.31	81.3	0.132	0.491	193	0.509
26	2	Low	33.52	125.1	0.173	0.459	193	0.509
30	2	Low	31.2	75.77	0.172	0.498	193	0.509
16	2	Low	34.54	122.22	0.18	0.519	193	0.509
18	2	Low	36.07	117.5	0.192	0.51	193	0.509
19	2	Low	36.89	106.35	0.148	0.484	193	0.509
18	2	Low	35.36	117.5	0.192	0.51	193	0.509
17	2	Low	34.59	76.56	0.188	0.509	193	0.509
2	2	Low	35.79	103.88	0.161	0.471	193	0.509
16	2	Low	36.97	122.22	0.18	0.519	193	0.509
20	2	Low	31.58	89.72	0.177	0.517	193	0.509
26	2	Low	36.18	125.1	0.173	0.459	193	0.509
23	2	Low	32.08	76.25	0.155	0.484	193	0.509
26	2	Low	39.39	125.1	0.173	0.459	193	0.509
2	2	Low	37.11	103.88	0.161	0.471	193	0.509
14	2	Low	38.01	110.71	0.181	0.467	193	0.509
21	2	Low	34.35	121.06	0.173	0.494	193	0.509

18	2	Low	34.08	117.5	0.192	0.51	193	0.509
16	2	Low	36.87	122.22	0.18	0.519	193	0.509
18	2	Low	37.87	117.5	0.192	0.51	193	0.509
20	2	Low	36.92	89.72	0.177	0.517	193	0.509
29	2	Low	36.35	98.82	0.18	0.471	193	0.509
27	2	Low	37.86	82.79	0.175	0.432	193	0.509
5	2	Low	35.35	97.7	0.163	0.517	193	0.509
11	2	Low	31.99	105.96	0.178	0.492	193	0.509
18	2	Low	40.63	117.5	0.192	0.51	193	0.509
26	2	Low	37	125.1	0.173	0.459	193	0.509
23	2	Low	36.84	76.25	0.155	0.484	193	0.509
21	2	Low	38.33	121.06	0.173	0.494	193	0.509
5	2	Low	38.45	97.7	0.163	0.517	193	0.509
21	2	Low	35.27	121.06	0.173	0.494	193	0.509
18	2	Low	35.87	117.5	0.192	0.51	193	0.509
18	2	Low	39.12	117.5	0.192	0.51	193	0.509
21	2	Low	34.27	121.06	0.173	0.494	193	0.509
11	2	Low	38.59	105.96	0.178	0.492	193	0.509
23	2	Low	30.49	76.25	0.155	0.484	193	0.509
18	2	Low	41.25	117.5	0.192	0.51	193	0.509
18	2	Low	37.66	117.5	0.192	0.51	193	0.509
26	2	Low	38.11	125.1	0.173	0.459	193	0.509
18	2	Low	42.7	117.5	0.192	0.51	193	0.509
18	2	Low	37.81	117.5	0.192	0.51	193	0.509
18	2	Low	38.94	117.5	0.192	0.51	193	0.509
2	2	Low	34.39	103.88	0.161	0.471	193	0.509
2	2	Low	37.9	103.88	0.161	0.471	193	0.509
16	2	Low	34.6	122.22	0.18	0.519	193	0.509
18	2	Low	40.54	117.5	0.192	0.51	193	0.509
14	2	Low	36.76	110.71	0.181	0.467	193	0.509

19	2	Low	36.1	106.35	0.148	0.484	193	0.509
21	2	Low	39.07	121.06	0.173	0.494	193	0.509
18	2	Low	35.23	117.5	0.192	0.51	193	0.509
23	2	Low	30.8	76.25	0.155	0.484	193	0.509
21	2	Low	38.84	121.06	0.173	0.494	193	0.509
22	2	Low	33.41	81.3	0.132	0.491	193	0.509
20	2	Low	37.81	89.72	0.177	0.517	193	0.509
1	2	Low	34.23	88.13	0.173	0.483	193	0.509
23	2	Low	33.67	76.25	0.155	0.484	193	0.509
21	2	Low	38.05	121.06	0.173	0.494	193	0.509
11	2	Low	32.36	105.96	0.178	0.492	193	0.509
8	2	Low	35.8	95.41	0.179	0.488	193	0.509
23	2	Low	26.92	76.25	0.155	0.484	193	0.509
18	2	Low	37.54	117.5	0.192	0.51	193	0.509
23	2	Low	27.52	76.25	0.155	0.484	193	0.509
23	2	Low	31.12	76.25	0.155	0.484	193	0.509
10	5	Low	40.98	85.28	0.172	0.499	198	1.629
24	5	Low	37.97	74.8	0.171	0.502	198	1.629
29	5	Low	42.51	98.82	0.18	0.471	198	1.629
29	5	Low	39.23	98.82	0.18	0.471	198	1.629
21	5	Low	46.71	121.06	0.173	0.494	198	1.629
5	5	Low	47.28	97.7	0.163	0.517	198	1.629
10	5	Low	42.19	85.28	0.172	0.499	198	1.629
28	5	Low	46.23	118	0.166	0.527	198	1.629
29	5	Low	45.6	98.82	0.18	0.471	198	1.629
14	5	Low	42.56	110.71	0.181	0.467	198	1.629
16	5	Low	43.54	122.22	0.18	0.519	198	1.629
21	5	Low	49.56	121.06	0.173	0.494	198	1.629
29	5	Low	42.48	98.82	0.18	0.471	198	1.629
29	5	Low	44.62	98.82	0.18	0.471	198	1.629

29	5	Low	43.11	98.82	0.18	0.471	198	1.629
24	5	Low	38.69	74.8	0.171	0.502	198	1.629
14	5	Low	41.89	110.71	0.181	0.467	198	1.629
16	5	Low	47.08	122.22	0.18	0.519	198	1.629
5	5	Low	41.66	97.7	0.163	0.517	198	1.629
16	5	Low	46.01	122.22	0.18	0.519	198	1.629
21	5	Low	51.36	121.06	0.173	0.494	198	1.629
24	5	Low	41.47	74.8	0.171	0.502	198	1.629
28	5	Low	51.87	118	0.166	0.527	198	1.629
16	5	Low	47.96	122.22	0.18	0.519	198	1.629
16	5	Low	48.1	122.22	0.18	0.519	198	1.629
28	5	Low	43.76	118	0.166	0.527	198	1.629
10	5	Low	40.32	85.28	0.172	0.499	198	1.629
3	5	Low	40.76	93.13	0.146	0.466	198	1.629
5	5	Low	41.76	97.7	0.163	0.517	198	1.629
5	5	Low	50.41	97.7	0.163	0.517	198	1.629
5	5	Low	43.23	97.7	0.163	0.517	198	1.629
21	5	Low	49.24	121.06	0.173	0.494	198	1.629
16	5	Low	46.84	122.22	0.18	0.519	198	1.629
28	5	Low	45.47	118	0.166	0.527	198	1.629
14	5	Low	43.4	110.71	0.181	0.467	198	1.629
21	5	Low	48.4	121.06	0.173	0.494	198	1.629
16	5	Low	40.04	122.22	0.18	0.519	198	1.629
16	5	Low	47.95	122.22	0.18	0.519	198	1.629
21	5	Low	44.6	121.06	0.173	0.494	198	1.629
6	5	Low	51.76	111.35	0.165	0.468	198	1.629
29	5	Low	41.06	98.82	0.18	0.471	198	1.629
16	5	Low	46.15	122.22	0.18	0.519	198	1.629
5	5	Low	47.83	97.7	0.163	0.517	198	1.629
21	5	Low	44.33	121.06	0.173	0.494	198	1.629

5	5	Low	42.64	97.7	0.163	0.517	198	1.629
3	5	Low	44.93	93.13	0.146	0.466	198	1.629
16	5	Low	46.84	122.22	0.18	0.519	198	1.629
28	5	Low	48.14	118	0.166	0.527	198	1.629
16	5	Low	50.67	122.22	0.18	0.519	198	1.629
29	5	Low	46.19	98.82	0.18	0.471	198	1.629
16	5	Low	41.17	122.22	0.18	0.519	198	1.629
16	5	Low	48.55	122.22	0.18	0.519	198	1.629
16	5	Low	49.51	122.22	0.18	0.519	198	1.629
10	5	Low	43.61	85.28	0.172	0.499	198	1.629
28	5	Low	46.97	118	0.166	0.527	198	1.629
16	5	Low	44.91	122.22	0.18	0.519	198	1.629
7	5	Low	41.46	91.94	0.166	0.493	198	1.629
16	5	Low	48.38	122.22	0.18	0.519	198	1.629
24	5	Low	38.69	74.8	0.171	0.502	198	1.629
7	5	Low	39.83	91.94	0.166	0.493	198	1.629
21	5	Low	46.1	121.06	0.173	0.494	198	1.629
3	5	Low	40.01	93.13	0.146	0.466	198	1.629
5	5	Low	46.28	97.7	0.163	0.517	198	1.629
28	5	Low	45.67	118	0.166	0.527	198	1.629
28	5	Low	46.58	118	0.166	0.527	198	1.629
16	5	Low	42.59	122.22	0.18	0.519	198	1.629
10	5	Low	42.58	85.28	0.172	0.499	198	1.629
21	5	Low	43.26	121.06	0.173	0.494	198	1.629
28	5	Low	42.22	118	0.166	0.527	198	1.629
3	5	Low	48.51	93.13	0.146	0.466	198	1.629
16	5	Low	43.85	122.22	0.18	0.519	198	1.629
16	5	Low	45.31	122.22	0.18	0.519	198	1.629
28	5	Low	43.84	118	0.166	0.527	198	1.629
16	5	Low	48.88	122.22	0.18	0.519	198	1.629

29	5	Low	47.84	98.82	0.18	0.471	198	1.629
16	5	Low	46.99	122.22	0.18	0.519	198	1.629
28	5	Low	45.68	118	0.166	0.527	198	1.629
5	5	Low	41.94	97.7	0.163	0.517	198	1.629
21	5	Low	47.91	121.06	0.173	0.494	198	1.629
24	5	Low	37.97	74.8	0.171	0.502	198	1.629
21	5	Low	49.06	121.06	0.173	0.494	198	1.629
7	5	Low	44.19	91.94	0.166	0.493	198	1.629
3	5	Low	47.38	93.13	0.146	0.466	198	1.629
28	5	Low	48.33	118	0.166	0.527	198	1.629
10	5	Low	44.15	85.28	0.172	0.499	198	1.629
28	5	Low	46.54	118	0.166	0.527	198	1.629
16	5	Low	44.1	122.22	0.18	0.519	198	1.629
29	5	Low	43.13	98.82	0.18	0.471	198	1.629
16	5	Low	51.44	122.22	0.18	0.519	198	1.629
16	5	Low	42.97	122.22	0.18	0.519	198	1.629
7	5	Low	46.2	91.94	0.166	0.493	198	1.629
5	5	Low	48.31	97.7	0.163	0.517	198	1.629
16	5	Low	41.75	122.22	0.18	0.519	198	1.629
5	5	Low	42.68	97.7	0.163	0.517	198	1.629
28	5	Low	42.18	118	0.166	0.527	198	1.629
16	5	Low	45.31	122.22	0.18	0.519	198	1.629
28	5	Low	47.39	118	0.166	0.527	198	1.629
16	5	Low	44	122.22	0.18	0.519	198	1.629
28	5	Low	47.86	118	0.166	0.527	198	1.629
16	5	Low	46.06	122.22	0.18	0.519	198	1.629
29	5	Low	36.43	98.82	0.18	0.471	198	1.629
6	5	Low	47.66	111.35	0.165	0.468	198	1.629
5	5	Low	42.34	97.7	0.163	0.517	198	1.629
5	5	Low	45.39	97.7	0.163	0.517	198	1.629

28	5	Low	52.79	118	0.166	0.527	198	1.629
5	5	Low	40.29	97.7	0.163	0.517	198	1.629
28	5	Low	49.17	118	0.166	0.527	198	1.629
24	5	Low	38.8	74.8	0.171	0.502	198	1.629
16	5	Low	50.05	122.22	0.18	0.519	198	1.629
21	5	Low	49.25	121.06	0.173	0.494	198	1.629
28	5	Low	48.32	118	0.166	0.527	198	1.629
21	5	Low	46.62	121.06	0.173	0.494	198	1.629
3	5	Low	44.92	93.13	0.146	0.466	198	1.629
21	5	Low	48.38	121.06	0.173	0.494	198	1.629
28	5	Low	48.29	118	0.166	0.527	198	1.629
28	5	Low	48.97	118	0.166	0.527	198	1.629
28	5	Low	46.44	118	0.166	0.527	198	1.629
21	5	Low	44.62	121.06	0.173	0.494	198	1.629
28	5	Low	50.71	118	0.166	0.527	198	1.629
4	5	Low	47.09	83.82	0.176	0.457	198	1.629
28	5	Low	44.6	118	0.166	0.527	198	1.629
16	5	Low	45.99	122.22	0.18	0.519	198	1.629
28	5	Low	48.56	118	0.166	0.527	198	1.629
21	5	Low	49.95	121.06	0.173	0.494	198	1.629
24	5	Low	37.08	74.8	0.171	0.502	198	1.629
5	5	Low	44.02	97.7	0.163	0.517	198	1.629
6	5	Low	49.09	111.35	0.165	0.468	198	1.629
16	5	Low	45.77	122.22	0.18	0.519	198	1.629
24	5	Low	38.54	74.8	0.171	0.502	198	1.629
21	5	Low	44.76	121.06	0.173	0.494	198	1.629
3	5	Low	45.59	93.13	0.146	0.466	198	1.629
7	5	Low	37.4	91.94	0.166	0.493	198	1.629
28	5	Low	44.18	118	0.166	0.527	198	1.629
28	5	Low	49.03	118	0.166	0.527	198	1.629

16	5	Low	44.86	122.22	0.18	0.519	198	1.629
24	5	Low	42.04	74.8	0.171	0.502	198	1.629
24	5	Low	41.9	74.8	0.171	0.502	198	1.629
28	5	Low	50.36	118	0.166	0.527	198	1.629
24	5	Low	39.92	74.8	0.171	0.502	198	1.629
29	5	Low	43.8	98.82	0.18	0.471	198	1.629
5	5	Low	41.27	97.7	0.163	0.517	198	1.629
16	5	Low	43.51	122.22	0.18	0.519	198	1.629
24	5	Low	39.38	74.8	0.171	0.502	198	1.629
24	5	Low	44.16	74.8	0.171	0.502	198	1.629
29	5	Low	44.74	98.82	0.18	0.471	198	1.629
28	5	Low	45.91	118	0.166	0.527	198	1.629
9	5	Low	49.31	80.99	0.172	0.484	198	1.629
24	5	Low	39.04	74.8	0.171	0.502	198	1.629
28	5	Low	42.71	118	0.166	0.527	198	1.629
21	5	Low	45.06	121.06	0.173	0.494	198	1.629
24	5	Low	39.49	74.8	0.171	0.502	198	1.629
28	5	Low	53.97	118	0.166	0.527	198	1.629
7	5	Low	46.54	91.94	0.166	0.493	198	1.629
3	5	Low	43.11	93.13	0.146	0.466	198	1.629
21	5	Low	53.37	121.06	0.173	0.494	198	1.629
16	5	Low	54.18	122.22	0.18	0.519	198	1.629
16	5	Low	47.45	122.22	0.18	0.519	198	1.629
4	5	Low	42.2	83.82	0.176	0.457	198	1.629
29	5	Low	44.41	98.82	0.18	0.471	198	1.629
24	5	Low	35.41	74.8	0.171	0.502	198	1.629
24	5	Low	39.68	74.8	0.171	0.502	198	1.629
24	5	Low	46.14	74.8	0.171	0.502	198	1.629
6	5	Low	44.54	111.35	0.165	0.468	198	1.629
20	5	Low	46.41	89.72	0.177	0.517	198	1.629

16	5	Low	48.05	122.22	0.18	0.519	198	1.629
16	5	Low	44.67	122.22	0.18	0.519	198	1.629
21	5	Low	44.91	121.06	0.173	0.494	198	1.629
18	6	Low	49.18	117.5	0.192	0.51	195	0.746
21	6	Low	45.57	121.06	0.173	0.494	195	0.746
8	6	Low	44.49	95.41	0.179	0.488	195	0.746
23	6	Low	31.9	76.25	0.155	0.484	195	0.746
1	6	Low	39.78	88.13	0.173	0.483	195	0.746
12	6	Low	46.9	96.67	0.17	0.477	195	0.746
21	6	Low	46.58	121.06	0.173	0.494	195	0.746
12	6	Low	40.28	96.67	0.17	0.477	195	0.746
22	6	Low	40	81.3	0.132	0.491	195	0.746
22	6	Low	41.87	81.3	0.132	0.491	195	0.746
14	6	Low	42.77	110.71	0.181	0.467	195	0.746
14	6	Low	42.9	110.71	0.181	0.467	195	0.746
22	6	Low	36.07	81.3	0.132	0.491	195	0.746
23	6	Low	42.87	76.25	0.155	0.484	195	0.746
26	6	Low	48.3	125.1	0.173	0.459	195	0.746
9	6	Low	36.93	80.99	0.172	0.484	195	0.746
22	6	Low	44.8	81.3	0.132	0.491	195	0.746
23	6	Low	37.63	76.25	0.155	0.484	195	0.746
11	6	Low	48.89	105.96	0.178	0.492	195	0.746
13	6	Low	44.99	77.78	0.139	0.507	195	0.746
7	6	Low	39.5	91.94	0.166	0.493	195	0.746
17	6	Low	42.77	76.56	0.188	0.509	195	0.746
10	6	Low	39.75	85.28	0.172	0.499	195	0.746
1	6	Low	45.32	88.13	0.173	0.483	195	0.746
2	6	Low	45.11	103.88	0.161	0.471	195	0.746
2	6	Low	40.76	103.88	0.161	0.471	195	0.746
1	6	Low	42.08	88.13	0.173	0.483	195	0.746

22	6	Low	40.94	81.3	0.132	0.491	195	0.746
11	6	Low	47.27	105.96	0.178	0.492	195	0.746
13	6	Low	46.45	77.78	0.139	0.507	195	0.746
18	6	Low	47.93	117.5	0.192	0.51	195	0.746
18	6	Low	45.23	117.5	0.192	0.51	195	0.746
1	6	Low	45.32	88.13	0.173	0.483	195	0.746
11	6	Low	40.07	105.96	0.178	0.492	195	0.746
2	6	Low	41.75	103.88	0.161	0.471	195	0.746
16	6	Low	43.73	122.22	0.18	0.519	195	0.746
8	6	Low	46.04	95.41	0.179	0.488	195	0.746
18	6	Low	48.62	117.5	0.192	0.51	195	0.746
22	6	Low	42.14	81.3	0.132	0.491	195	0.746
18	6	Low	47.58	117.5	0.192	0.51	195	0.746
30	6	Low	42.21	75.77	0.172	0.498	195	0.746
23	6	Low	43.65	76.25	0.155	0.484	195	0.746
14	6	Low	48.11	110.71	0.181	0.467	195	0.746
30	6	Low	36.42	75.77	0.172	0.498	195	0.746
9	6	Low	39.76	80.99	0.172	0.484	195	0.746
23	6	Low	40.14	76.25	0.155	0.484	195	0.746
23	6	Low	37.89	76.25	0.155	0.484	195	0.746
11	6	Low	45.54	105.96	0.178	0.492	195	0.746
18	6	Low	39	117.5	0.192	0.51	195	0.746
26	6	Low	45.24	125.1	0.173	0.459	195	0.746
19	6	Low	41.94	106.35	0.148	0.484	195	0.746
23	6	Low	36.63	76.25	0.155	0.484	195	0.746
19	6	Low	42.02	106.35	0.148	0.484	195	0.746
21	6	Low	48.36	121.06	0.173	0.494	195	0.746
23	6	Low	40.81	76.25	0.155	0.484	195	0.746
18	6	Low	46.42	117.5	0.192	0.51	195	0.746
12	6	Low	42.24	96.67	0.17	0.477	195	0.746

26	6	Low	47	125.1	0.173	0.459	195	0.746
18	6	Low	49.06	117.5	0.192	0.51	195	0.746
11	6	Low	44.33	105.96	0.178	0.492	195	0.746
2	6	Low	45.48	103.88	0.161	0.471	195	0.746
8	6	Low	40.65	95.41	0.179	0.488	195	0.746
15	6	Low	43.41	85.31	0.168	0.486	195	0.746
19	6	Low	50.61	106.35	0.148	0.484	195	0.746
2	6	Low	40.4	103.88	0.161	0.471	195	0.746
17	6	Low	44.18	76.56	0.188	0.509	195	0.746
15	6	Low	41.15	85.31	0.168	0.486	195	0.746
18	6	Low	50.79	117.5	0.192	0.51	195	0.746
1	6	Low	42.45	88.13	0.173	0.483	195	0.746
10	6	Low	42.64	85.28	0.172	0.499	195	0.746
23	6	Low	41.67	76.25	0.155	0.484	195	0.746
17	6	Low	47.15	76.56	0.188	0.509	195	0.746
11	6	Low	49.1	105.96	0.178	0.492	195	0.746
9	6	Low	39.39	80.99	0.172	0.484	195	0.746
10	6	Low	42.18	85.28	0.172	0.499	195	0.746
22	6	Low	43.54	81.3	0.132	0.491	195	0.746
2	6	Low	44.98	103.88	0.161	0.471	195	0.746
11	6	Low	45.62	105.96	0.178	0.492	195	0.746
4	6	Low	38.86	83.82	0.176	0.457	195	0.746
13	6	Low	40.98	77.78	0.139	0.507	195	0.746
19	6	Low	44.66	106.35	0.148	0.484	195	0.746
19	6	Low	42.79	106.35	0.148	0.484	195	0.746
20	6	Low	40.41	89.72	0.177	0.517	195	0.746
18	6	Low	46.59	117.5	0.192	0.51	195	0.746
30	6	Low	43.1	75.77	0.172	0.498	195	0.746
9	6	Low	37.92	80.99	0.172	0.484	195	0.746
13	6	Low	41.53	77.78	0.139	0.507	195	0.746

2	6	Low	46.53	103.88	0.161	0.471	195	0.746
26	6	Low	41.63	125.1	0.173	0.459	195	0.746
11	6	Low	42.79	105.96	0.178	0.492	195	0.746
23	6	Low	40.32	76.25	0.155	0.484	195	0.746
2	6	Low	47.31	103.88	0.161	0.471	195	0.746
7	6	Low	43.09	91.94	0.166	0.493	195	0.746
9	6	Low	35.64	80.99	0.172	0.484	195	0.746
2	6	Low	44.82	103.88	0.161	0.471	195	0.746
18	6	Low	46.99	117.5	0.192	0.51	195	0.746
19	6	Low	45.31	106.35	0.148	0.484	195	0.746
14	6	Low	45.98	110.71	0.181	0.467	195	0.746
19	6	Low	43.96	106.35	0.148	0.484	195	0.746
20	6	Low	48.52	89.72	0.177	0.517	195	0.746
7	6	Low	42.61	91.94	0.166	0.493	195	0.746
10	6	Low	44.2	85.28	0.172	0.499	195	0.746
10	6	Low	41.77	85.28	0.172	0.499	195	0.746
30	6	Low	42.97	75.77	0.172	0.498	195	0.746
2	6	Low	41.98	103.88	0.161	0.471	195	0.746
15	6	Low	43.47	85.31	0.168	0.486	195	0.746
5	6	Low	44.36	97.7	0.163	0.517	195	0.746
19	6	Low	45	106.35	0.148	0.484	195	0.746
9	6	Low	41.38	80.99	0.172	0.484	195	0.746
9	6	Low	43.58	80.99	0.172	0.484	195	0.746
5	6	Low	42.78	97.7	0.163	0.517	195	0.746
10	6	Low	47.38	85.28	0.172	0.499	195	0.746
20	6	Low	48.69	89.72	0.177	0.517	195	0.746
24	6	Low	37.95	74.8	0.171	0.502	195	0.746
22	6	Low	43.79	81.3	0.132	0.491	195	0.746
29	6	Low	45.47	98.82	0.18	0.471	195	0.746
20	6	Low	47.36	89.72	0.177	0.517	195	0.746

21	6	Low	52.23	121.06	0.173	0.494	195	0.746
11	6	Low	47.83	105.96	0.178	0.492	195	0.746
9	6	Low	45.12	80.99	0.172	0.484	195	0.746
18	6	Low	48.95	117.5	0.192	0.51	195	0.746
9	6	Low	44.63	80.99	0.172	0.484	195	0.746
9	6	Low	42.29	80.99	0.172	0.484	195	0.746
19	6	Low	47.01	106.35	0.148	0.484	195	0.746
4	6	Low	36.85	83.82	0.176	0.457	195	0.746
30	6	Low	38.88	75.77	0.172	0.498	195	0.746
24	6	Low	40.7	74.8	0.171	0.502	195	0.746
26	6	Low	45.94	125.1	0.173	0.459	195	0.746
9	6	Low	42.97	80.99	0.172	0.484	195	0.746
15	6	Low	46.98	85.31	0.168	0.486	195	0.746
9	6	Low	39.71	80.99	0.172	0.484	195	0.746
26	6	Low	47.73	125.1	0.173	0.459	195	0.746
8	6	Low	45.91	95.41	0.179	0.488	195	0.746
19	6	Low	45.9	106.35	0.148	0.484	195	0.746
14	6	Low	47.23	110.71	0.181	0.467	195	0.746
12	6	Low	44.05	96.67	0.17	0.477	195	0.746
30	6	Low	35.01	75.77	0.172	0.498	195	0.746
1	6	Low	42.18	88.13	0.173	0.483	195	0.746
10	6	Low	42.92	85.28	0.172	0.499	195	0.746
7	6	Low	39.68	91.94	0.166	0.493	195	0.746
6	6	Low	44.91	111.35	0.165	0.468	195	0.746
21	6	Low	47.04	121.06	0.173	0.494	195	0.746
11	6	Low	47.51	105.96	0.178	0.492	195	0.746
26	6	Low	49.13	125.1	0.173	0.459	195	0.746
7	6	Low	40.95	91.94	0.166	0.493	195	0.746
23	6	Low	44.69	76.25	0.155	0.484	195	0.746
30	6	Low	40.91	75.77	0.172	0.498	195	0.746

14	6	Low	49.14	110.71	0.181	0.467	195	0.746
9	6	Low	37.49	80.99	0.172	0.484	195	0.746
5	6	Low	40.03	97.7	0.163	0.517	195	0.746
30	6	Low	40.83	75.77	0.172	0.498	195	0.746
23	6	Low	43.96	76.25	0.155	0.484	195	0.746
30	6	Low	37.36	75.77	0.172	0.498	195	0.746
18	6	Low	49.9	117.5	0.192	0.51	195	0.746
24	8	Low	49.07	74.8	0.171	0.502	206	0.296
12	8	Low	48.32	96.67	0.17	0.477	206	0.296
10	8	Low	50.04	85.28	0.172	0.499	206	0.296
18	8	Low	56.49	117.5	0.192	0.51	206	0.296
27	8	Low	46.34	82.79	0.175	0.432	206	0.296
12	8	Low	50.98	96.67	0.17	0.477	206	0.296
20	8	Low	53.33	89.72	0.177	0.517	206	0.296
27	8	Low	56.22	82.79	0.175	0.432	206	0.296
15	8	Low	52.65	85.31	0.168	0.486	206	0.296
15	8	Low	51.13	85.31	0.168	0.486	206	0.296
18	8	Low	51.85	117.5	0.192	0.51	206	0.296
18	8	Low	57.77	117.5	0.192	0.51	206	0.296
23	8	Low	48.96	76.25	0.155	0.484	206	0.296
15	8	Low	49.45	85.31	0.168	0.486	206	0.296
19	8	Low	48.6	106.35	0.148	0.484	206	0.296
15	8	Low	52.85	85.31	0.168	0.486	206	0.296
10	8	Low	52.2	85.28	0.172	0.499	206	0.296
17	8	Low	50	76.56	0.188	0.509	206	0.296
7	8	Low	47.61	91.94	0.166	0.493	206	0.296
23	8	Low	49.29	76.25	0.155	0.484	206	0.296
9	8	Low	45.75	80.99	0.172	0.484	206	0.296
18	8	Low	55.31	117.5	0.192	0.51	206	0.296
24	8	Low	46.35	74.8	0.171	0.502	206	0.296

12	8	Low	51.47	96.67	0.17	0.477	206	0.296
23	8	Low	48.35	76.25	0.155	0.484	206	0.296
13	8	Low	55.41	77.78	0.139	0.507	206	0.296
7	8	Low	50.1	91.94	0.166	0.493	206	0.296
6	8	Low	49.36	111.35	0.165	0.468	206	0.296
11	8	Low	50.66	105.96	0.178	0.492	206	0.296
6	8	Low	51.14	111.35	0.165	0.468	206	0.296
11	8	Low	46.05	105.96	0.178	0.492	206	0.296
24	8	Low	47.02	74.8	0.171	0.502	206	0.296
27	8	Low	50.81	82.79	0.175	0.432	206	0.296
27	8	Low	50.19	82.79	0.175	0.432	206	0.296
20	8	Low	56.34	89.72	0.177	0.517	206	0.296
21	8	Low	51.16	121.06	0.173	0.494	206	0.296
12	8	Low	50.13	96.67	0.17	0.477	206	0.296
19	8	Low	58.46	106.35	0.148	0.484	206	0.296
12	8	Low	50.57	96.67	0.17	0.477	206	0.296
8	8	Low	58.27	95.41	0.179	0.488	206	0.296
12	8	Low	53.75	96.67	0.17	0.477	206	0.296
23	8	Low	50.7	76.25	0.155	0.484	206	0.296
7	8	Low	46.99	91.94	0.166	0.493	206	0.296
26	8	Low	57.84	125.1	0.173	0.459	206	0.296
19	8	Low	53.46	106.35	0.148	0.484	206	0.296
22	8	Low	46.45	81.3	0.132	0.491	206	0.296
9	8	Low	46.33	80.99	0.172	0.484	206	0.296
5	8	Low	56.23	97.7	0.163	0.517	206	0.296
5	8	Low	54.94	97.7	0.163	0.517	206	0.296
9	8	Low	47.29	80.99	0.172	0.484	206	0.296
23	8	Low	50.8	76.25	0.155	0.484	206	0.296
12	8	Low	47.33	96.67	0.17	0.477	206	0.296
7	8	Low	51.1	91.94	0.166	0.493	206	0.296

19	8	Low	49.38	106.35	0.148	0.484	206	0.296
9	8	Low	46.63	80.99	0.172	0.484	206	0.296
12	8	Low	50.59	96.67	0.17	0.477	206	0.296
20	8	Low	54.75	89.72	0.177	0.517	206	0.296
24	8	Low	51.77	74.8	0.171	0.502	206	0.296
27	8	Low	48.53	82.79	0.175	0.432	206	0.296
12	8	Low	53.12	96.67	0.17	0.477	206	0.296
18	8	Low	45.7	117.5	0.192	0.51	206	0.296
18	8	Low	51.31	117.5	0.192	0.51	206	0.296
22	8	Low	40.71	81.3	0.132	0.491	206	0.296
15	8	Low	50.49	85.31	0.168	0.486	206	0.296
1	8	Low	51.68	88.13	0.173	0.483	206	0.296
11	8	Low	57.88	105.96	0.178	0.492	206	0.296
17	8	Low	50.36	76.56	0.188	0.509	206	0.296
6	8	Low	54.22	111.35	0.165	0.468	206	0.296
11	8	Low	52.13	105.96	0.178	0.492	206	0.296
8	8	Low	53.53	95.41	0.179	0.488	206	0.296
12	8	Low	52.8	96.67	0.17	0.477	206	0.296
6	8	Low	40.1	111.35	0.165	0.468	206	0.296
18	8	Low	53.15	117.5	0.192	0.51	206	0.296
15	8	Low	50.46	85.31	0.168	0.486	206	0.296
20	8	Low	57.13	89.72	0.177	0.517	206	0.296
29	8	Low	49	98.82	0.18	0.471	206	0.296
24	8	Low	46.86	74.8	0.171	0.502	206	0.296
14	8	Low	50.33	110.71	0.181	0.467	206	0.296
6	8	Low	50.25	111.35	0.165	0.468	206	0.296
20	8	Low	51.22	89.72	0.177	0.517	206	0.296
9	8	Low	47.17	80.99	0.172	0.484	206	0.296
6	8	Low	49.29	111.35	0.165	0.468	206	0.296
2	8	Low	52.4	103.88	0.161	0.471	206	0.296

19	8	Low	50.92	106.35	0.148	0.484	206	0.296
23	3	High	60.26	76.25	0.155	0.484	205	0.746
10	3	High	61.67	85.28	0.172	0.499	205	0.746
19	3	High	59.17	106.35	0.148	0.484	205	0.746
30	3	High	48.93	75.77	0.172	0.498	205	0.746
9	3	High	52.62	80.99	0.172	0.484	205	0.746
17	3	High	57.31	76.56	0.188	0.509	205	0.746
14	3	High	64.56	110.71	0.181	0.467	205	0.746
12	3	High	58.27	96.67	0.17	0.477	205	0.746
27	3	High	57.85	82.79	0.175	0.432	205	0.746
7	3	High	56.98	91.94	0.166	0.493	205	0.746
20	3	High	64.11	89.72	0.177	0.517	205	0.746
23	3	High	62.31	76.25	0.155	0.484	205	0.746
7	3	High	56.55	91.94	0.166	0.493	205	0.746
30	3	High	53.8	75.77	0.172	0.498	205	0.746
9	3	High	58.43	80.99	0.172	0.484	205	0.746
1	3	High	62.9	88.13	0.173	0.483	205	0.746
27	3	High	64.22	82.79	0.175	0.432	205	0.746
9	3	High	56.9	80.99	0.172	0.484	205	0.746
30	3	High	59.2	75.77	0.172	0.498	205	0.746
17	3	High	63.84	76.56	0.188	0.509	205	0.746
23	3	High	55.23	76.25	0.155	0.484	205	0.746
10	3	High	59.74	85.28	0.172	0.499	205	0.746
10	3	High	60.76	85.28	0.172	0.499	205	0.746
17	3	High	59.88	76.56	0.188	0.509	205	0.746
5	3	High	62.5	97.7	0.163	0.517	205	0.746
2	3	High	44.79	103.88	0.161	0.471	205	0.746
20	3	High	66.54	89.72	0.177	0.517	205	0.746
27	3	High	57.29	82.79	0.175	0.432	205	0.746
30	3	High	54.29	75.77	0.172	0.498	205	0.746

18	3	High	64.62	117.5	0.192	0.51	205	0.746
9	3	High	55.89	80.99	0.172	0.484	205	0.746
9	3	High	55.34	80.99	0.172	0.484	205	0.746
11	3	High	57.38	105.96	0.178	0.492	205	0.746
2	3	High	55.56	103.88	0.161	0.471	205	0.746
23	3	High	55.67	76.25	0.155	0.484	205	0.746
29	3	High	59.35	98.82	0.18	0.471	205	0.746
2	3	High	58.05	103.88	0.161	0.471	205	0.746
30	3	High	55.47	75.77	0.172	0.498	205	0.746
11	3	High	57.23	105.96	0.178	0.492	205	0.746
3	3	High	55.93	93.13	0.146	0.466	205	0.746
9	3	High	62.64	80.99	0.172	0.484	205	0.746
12	3	High	59.28	96.67	0.17	0.477	205	0.746
18	3	High	70.2	117.5	0.192	0.51	205	0.746
23	3	High	57.31	76.25	0.155	0.484	205	0.746
23	3	High	56.89	76.25	0.155	0.484	205	0.746
10	3	High	58.03	85.28	0.172	0.499	205	0.746
9	3	High	58.61	80.99	0.172	0.484	205	0.746
20	3	High	60.29	89.72	0.177	0.517	205	0.746
23	3	High	55.04	76.25	0.155	0.484	205	0.746
18	3	High	66.87	117.5	0.192	0.51	205	0.746
30	3	High	56.61	75.77	0.172	0.498	205	0.746
12	3	High	58.45	96.67	0.17	0.477	205	0.746
23	3	High	59.9	76.25	0.155	0.484	205	0.746
23	3	High	61.21	76.25	0.155	0.484	205	0.746
23	3	High	59.11	76.25	0.155	0.484	205	0.746
2	3	High	60.35	103.88	0.161	0.471	205	0.746
26	3	High	64.86	125.1	0.173	0.459	205	0.746
27	3	High	61.35	82.79	0.175	0.432	205	0.746
9	3	High	54.79	80.99	0.172	0.484	205	0.746

21	3	High	63.54	121.06	0.173	0.494	205	0.746
27	3	High	56.63	82.79	0.175	0.432	205	0.746
10	3	High	56.77	85.28	0.172	0.499	205	0.746
8	3	High	63.66	95.41	0.179	0.488	205	0.746
19	3	High	55.93	106.35	0.148	0.484	205	0.746
15	3	High	59.88	85.31	0.168	0.486	205	0.746
2	3	High	61.1	103.88	0.161	0.471	205	0.746
10	3	High	58.02	85.28	0.172	0.499	205	0.746
9	3	High	57.94	80.99	0.172	0.484	205	0.746
9	3	High	54.35	80.99	0.172	0.484	205	0.746
2	3	High	63.14	103.88	0.161	0.471	205	0.746
27	3	High	53.78	82.79	0.175	0.432	205	0.746
18	3	High	61.43	117.5	0.192	0.51	205	0.746
17	3	High	59.41	76.56	0.188	0.509	205	0.746
10	3	High	57.29	85.28	0.172	0.499	205	0.746
12	3	High	61.03	96.67	0.17	0.477	205	0.746
1	3	High	63.52	88.13	0.173	0.483	205	0.746
20	3	High	62.89	89.72	0.177	0.517	205	0.746
17	3	High	61.07	76.56	0.188	0.509	205	0.746
13	3	High	61.17	77.78	0.139	0.507	205	0.746
23	3	High	55.5	76.25	0.155	0.484	205	0.746
19	3	High	62.86	106.35	0.148	0.484	205	0.746
9	3	High	59.66	80.99	0.172	0.484	205	0.746
15	3	High	59.2	85.31	0.168	0.486	205	0.746
7	3	High	59.96	91.94	0.166	0.493	205	0.746
24	3	High	55.93	74.8	0.171	0.502	205	0.746
2	3	High	62.59	103.88	0.161	0.471	205	0.746
20	3	High	61	89.72	0.177	0.517	205	0.746
23	3	High	56.42	76.25	0.155	0.484	205	0.746
22	3	High	59.71	81.3	0.132	0.491	205	0.746

10	3	High	56.35	85.28	0.172	0.499	205	0.746
15	3	High	62.39	85.31	0.168	0.486	205	0.746
11	3	High	55.32	105.96	0.178	0.492	205	0.746
26	3	High	63.04	125.1	0.173	0.459	205	0.746
23	3	High	52.39	76.25	0.155	0.484	205	0.746
11	3	High	57.77	105.96	0.178	0.492	205	0.746
10	3	High	60.66	85.28	0.172	0.499	205	0.746
7	3	High	60.77	91.94	0.166	0.493	205	0.746
23	3	High	57.78	76.25	0.155	0.484	205	0.746
23	3	High	56.15	76.25	0.155	0.484	205	0.746
2	3	High	59.14	103.88	0.161	0.471	205	0.746
27	3	High	58.35	82.79	0.175	0.432	205	0.746
18	3	High	59.34	117.5	0.192	0.51	205	0.746
20	3	High	59.49	89.72	0.177	0.517	205	0.746
18	4	High	46.24	117.5	0.192	0.51	194	0.61
18	4	High	48.79	117.5	0.192	0.51	194	0.61
10	4	High	42.49	85.28	0.172	0.499	194	0.61
2	4	High	37.73	103.88	0.161	0.471	194	0.61
26	4	High	41.57	125.1	0.173	0.459	194	0.61
18	4	High	49.79	117.5	0.192	0.51	194	0.61
18	4	High	48.94	117.5	0.192	0.51	194	0.61
12	4	High	41.34	96.67	0.17	0.477	194	0.61
18	4	High	42.78	117.5	0.192	0.51	194	0.61
18	4	High	48.59	117.5	0.192	0.51	194	0.61
18	4	High	46.41	117.5	0.192	0.51	194	0.61
2	4	High	40.71	103.88	0.161	0.471	194	0.61
18	4	High	48.92	117.5	0.192	0.51	194	0.61
2	4	High	37.24	103.88	0.161	0.471	194	0.61
12	4	High	46.93	96.67	0.17	0.477	194	0.61
19	4	High	44.72	106.35	0.148	0.484	194	0.61

9	4	High	38.21	80.99	0.172	0.484	194	0.61
12	4	High	39.86	96.67	0.17	0.477	194	0.61
11	4	High	40.96	105.96	0.178	0.492	194	0.61
21	4	High	41.41	121.06	0.173	0.494	194	0.61
14	4	High	48.98	110.71	0.181	0.467	194	0.61
15	4	High	42.75	85.31	0.168	0.486	194	0.61
12	4	High	45.31	96.67	0.17	0.477	194	0.61
9	4	High	40.49	80.99	0.172	0.484	194	0.61
12	4	High	41.57	96.67	0.17	0.477	194	0.61
12	4	High	43.73	96.67	0.17	0.477	194	0.61
12	4	High	42.25	96.67	0.17	0.477	194	0.61
6	4	High	44.78	111.35	0.165	0.468	194	0.61
19	4	High	46.74	106.35	0.148	0.484	194	0.61
11	4	High	46.83	105.96	0.178	0.492	194	0.61
11	4	High	36.01	105.96	0.178	0.492	194	0.61
27	4	High	41.97	82.79	0.175	0.432	194	0.61
19	4	High	50.76	106.35	0.148	0.484	194	0.61
17	4	High	41.06	76.56	0.188	0.509	194	0.61
5	4	High	46.49	97.7	0.163	0.517	194	0.61
21	4	High	45.88	121.06	0.173	0.494	194	0.61
26	4	High	37.77	125.1	0.173	0.459	194	0.61
12	4	High	43.9	96.67	0.17	0.477	194	0.61
11	4	High	43.38	105.96	0.178	0.492	194	0.61
18	4	High	49.18	117.5	0.192	0.51	194	0.61
2	4	High	37.1	103.88	0.161	0.471	194	0.61
18	4	High	51.05	117.5	0.192	0.51	194	0.61
18	4	High	47.11	117.5	0.192	0.51	194	0.61
2	4	High	44	103.88	0.161	0.471	194	0.61
24	4	High	41.39	74.8	0.171	0.502	194	0.61
18	4	High	49.93	117.5	0.192	0.51	194	0.61

20	4	High	44.7	89.72	0.177	0.517	194	0.61
17	4	High	43.95	76.56	0.188	0.509	194	0.61
18	4	High	51.59	117.5	0.192	0.51	194	0.61
6	4	High	46.13	111.35	0.165	0.468	194	0.61
11	4	High	38.19	105.96	0.178	0.492	194	0.61
20	4	High	41.43	89.72	0.177	0.517	194	0.61
2	4	High	44.61	103.88	0.161	0.471	194	0.61
2	4	High	44.32	103.88	0.161	0.471	194	0.61
6	4	High	43.48	111.35	0.165	0.468	194	0.61
27	4	High	39.42	82.79	0.175	0.432	194	0.61
12	4	High	44.97	96.67	0.17	0.477	194	0.61
19	4	High	43.07	106.35	0.148	0.484	194	0.61
7	4	High	40.66	91.94	0.166	0.493	194	0.61
22	4	High	38.32	81.3	0.132	0.491	194	0.61
17	4	High	41.97	76.56	0.188	0.509	194	0.61
18	4	High	47.96	117.5	0.192	0.51	194	0.61
12	4	High	43.78	96.67	0.17	0.477	194	0.61
21	4	High	46.63	121.06	0.173	0.494	194	0.61
18	4	High	40.94	117.5	0.192	0.51	194	0.61
20	4	High	45.51	89.72	0.177	0.517	194	0.61
19	4	High	43.78	106.35	0.148	0.484	194	0.61
23	4	High	42.08	76.25	0.155	0.484	194	0.61
18	4	High	44.71	117.5	0.192	0.51	194	0.61
20	4	High	44.96	89.72	0.177	0.517	194	0.61
9	4	High	41.95	80.99	0.172	0.484	194	0.61
18	4	High	43.42	117.5	0.192	0.51	194	0.61
18	4	High	46.88	117.5	0.192	0.51	194	0.61
1	4	High	36.77	88.13	0.173	0.483	194	0.61
7	4	High	41.21	91.94	0.166	0.493	194	0.61
19	4	High	36.49	106.35	0.148	0.484	194	0.61

7	4	High	42.5	91.94	0.166	0.493	194	0.61
9	4	High	38.28	80.99	0.172	0.484	194	0.61
23	4	High	41.84	76.25	0.155	0.484	194	0.61
12	4	High	36.44	96.67	0.17	0.477	194	0.61
9	4	High	39.7	80.99	0.172	0.484	194	0.61
15	4	High	39.29	85.31	0.168	0.486	194	0.61
19	4	High	43.11	106.35	0.148	0.484	194	0.61
23	4	High	40.96	76.25	0.155	0.484	194	0.61
20	4	High	44.05	89.72	0.177	0.517	194	0.61
18	4	High	47.93	117.5	0.192	0.51	194	0.61
24	4	High	43.89	74.8	0.171	0.502	194	0.61
26	4	High	44.07	125.1	0.173	0.459	194	0.61
3	4	High	42.8	93.13	0.146	0.466	194	0.61
20	4	High	42.1	89.72	0.177	0.517	194	0.61
21	4	High	45.05	121.06	0.173	0.494	194	0.61
18	4	High	51.36	117.5	0.192	0.51	194	0.61
24	4	High	39.84	74.8	0.171	0.502	194	0.61
18	4	High	44.29	117.5	0.192	0.51	194	0.61
2	4	High	43.77	103.88	0.161	0.471	194	0.61
30	4	High	38.4	75.77	0.172	0.498	194	0.61
12	4	High	43.53	96.67	0.17	0.477	194	0.61
26	4	High	36.84	125.1	0.173	0.459	194	0.61
26	4	High	47.11	125.1	0.173	0.459	194	0.61
12	4	High	44.41	96.67	0.17	0.477	194	0.61
26	4	High	50.62	125.1	0.173	0.459	194	0.61
12	4	High	41.73	96.67	0.17	0.477	194	0.61
30	4	High	39.05	75.77	0.172	0.498	194	0.61
14	4	High	40.1	110.71	0.181	0.467	194	0.61
7	4	High	42.72	91.94	0.166	0.493	194	0.61
10	4	High	39.86	85.28	0.172	0.499	194	0.61

26	4	High	33.13	125.1	0.173	0.459	194	0.61
2	4	High	37.76	103.88	0.161	0.471	194	0.61
18	4	High	42.74	117.5	0.192	0.51	194	0.61
19	4	High	40.32	106.35	0.148	0.484	194	0.61
6	4	High	45.14	111.35	0.165	0.468	194	0.61
18	4	High	40.82	117.5	0.192	0.51	194	0.61
9	4	High	39.8	80.99	0.172	0.484	194	0.61
1	4	High	40.52	88.13	0.173	0.483	194	0.61
2	4	High	47.83	103.88	0.161	0.471	194	0.61
18	4	High	41.26	117.5	0.192	0.51	194	0.61
10	4	High	40.9	85.28	0.172	0.499	194	0.61
15	4	High	40.72	85.31	0.168	0.486	194	0.61
2	4	High	41.37	103.88	0.161	0.471	194	0.61
18	4	High	49.04	117.5	0.192	0.51	194	0.61
18	4	High	44.01	117.5	0.192	0.51	194	0.61
24	4	High	39.39	74.8	0.171	0.502	194	0.61
26	4	High	47.77	125.1	0.173	0.459	194	0.61
21	4	High	46.33	121.06	0.173	0.494	194	0.61
13	4	High	37.75	77.78	0.139	0.507	194	0.61
21	4	High	46.07	121.06	0.173	0.494	194	0.61
14	4	High	44.25	110.71	0.181	0.467	194	0.61
11	4	High	46.5	105.96	0.178	0.492	194	0.61
20	4	High	48.99	89.72	0.177	0.517	194	0.61
2	4	High	40.74	103.88	0.161	0.471	194	0.61
18	4	High	37.14	117.5	0.192	0.51	194	0.61
18	4	High	47.16	117.5	0.192	0.51	194	0.61
21	4	High	47.34	121.06	0.173	0.494	194	0.61
18	4	High	48.32	117.5	0.192	0.51	194	0.61
11	4	High	43.28	105.96	0.178	0.492	194	0.61
19	4	High	46.07	106.35	0.148	0.484	194	0.61

2	4	High	44.02	103.88	0.161	0.471	194	0.61
14	4	High	48.62	110.71	0.181	0.467	194	0.61
19	4	High	38.05	106.35	0.148	0.484	194	0.61
16	4	High	44.65	122.22	0.18	0.519	194	0.61
19	4	High	46.17	106.35	0.148	0.484	194	0.61
29	7	High	54.71	98.82	0.18	0.471	203	1.01
24	7	High	45.94	74.8	0.171	0.502	203	1.01
14	7	High	58.12	110.71	0.181	0.467	203	1.01
12	7	High	53.04	96.67	0.17	0.477	203	1.01
20	7	High	59.27	89.72	0.177	0.517	203	1.01
21	7	High	51.57	121.06	0.173	0.494	203	1.01
4	7	High	52.65	83.82	0.176	0.457	203	1.01
6	7	High	56.06	111.35	0.165	0.468	203	1.01
30	7	High	47.99	75.77	0.172	0.498	203	1.01
21	7	High	53.92	121.06	0.173	0.494	203	1.01
7	7	High	51.89	91.94	0.166	0.493	203	1.01
15	7	High	51.93	85.31	0.168	0.486	203	1.01
17	7	High	53.88	76.56	0.188	0.509	203	1.01
7	7	High	49.06	91.94	0.166	0.493	203	1.01
17	7	High	50.58	76.56	0.188	0.509	203	1.01
24	7	High	50.33	74.8	0.171	0.502	203	1.01
4	7	High	52.41	83.82	0.176	0.457	203	1.01
16	7	High	52.38	122.22	0.18	0.519	203	1.01
19	7	High	51.19	106.35	0.148	0.484	203	1.01
12	7	High	53.73	96.67	0.17	0.477	203	1.01
4	7	High	49.08	83.82	0.176	0.457	203	1.01
29	7	High	52.64	98.82	0.18	0.471	203	1.01
15	7	High	47.33	85.31	0.168	0.486	203	1.01
4	7	High	54.1	83.82	0.176	0.457	203	1.01
29	7	High	49.19	98.82	0.18	0.471	203	1.01

28	7	High	58.3	118	0.166	0.527	203	1.01
19	7	High	52.36	106.35	0.148	0.484	203	1.01
24	7	High	48.33	74.8	0.171	0.502	203	1.01
29	7	High	50.64	98.82	0.18	0.471	203	1.01
20	7	High	51.58	89.72	0.177	0.517	203	1.01
24	7	High	46.61	74.8	0.171	0.502	203	1.01
24	7	High	48.98	74.8	0.171	0.502	203	1.01
3	7	High	44.6	93.13	0.146	0.466	203	1.01
29	7	High	49.95	98.82	0.18	0.471	203	1.01
16	7	High	57.14	122.22	0.18	0.519	203	1.01
24	7	High	52.88	74.8	0.171	0.502	203	1.01
10	7	High	51.07	85.28	0.172	0.499	203	1.01
1	7	High	55.74	88.13	0.173	0.483	203	1.01
4	7	High	50.98	83.82	0.176	0.457	203	1.01
24	7	High		74.8	0.171	0.502	203	1.01
19	7	High	58.43	106.35	0.148	0.484	203	1.01
24	7	High	49.78	74.8	0.171	0.502	203	1.01
24	7	High	48.09	74.8	0.171	0.502	203	1.01
24	7	High	49.62	74.8	0.171	0.502	203	1.01
24	7	High	49.1	74.8	0.171	0.502	203	1.01
12	7	High	53.85	96.67	0.17	0.477	203	1.01
8	7	High	56.39	95.41	0.179	0.488	203	1.01
10	7	High	51.35	85.28	0.172	0.499	203	1.01
16	7	High	52.65	122.22	0.18	0.519	203	1.01
6	7	High	57.07	111.35	0.165	0.468	203	1.01
5	7	High	51.39	97.7	0.163	0.517	203	1.01
29	7	High	48.52	98.82	0.18	0.471	203	1.01
16	7	High	58.31	122.22	0.18	0.519	203	1.01
29	7	High	53.81	98.82	0.18	0.471	203	1.01
18	7	High	56.79	117.5	0.192	0.51	203	1.01

4	7	High	50.65	83.82	0.176	0.457	203	1.01
24	7	High	48.72	74.8	0.171	0.502	203	1.01
21	7	High	57.99	121.06	0.173	0.494	203	1.01
7	7	High	38.45	91.94	0.166	0.493	203	1.01
15	7	High	51.93	85.31	0.168	0.486	203	1.01
11	7	High	51.24	105.96	0.178	0.492	203	1.01
3	7	High	52.78	93.13	0.146	0.466	203	1.01
15	7	High	49.6	85.31	0.168	0.486	203	1.01
20	7	High	51.05	89.72	0.177	0.517	203	1.01
12	7	High	52.36	96.67	0.17	0.477	203	1.01
7	7	High	49.31	91.94	0.166	0.493	203	1.01
11	7	High	53.45	105.96	0.178	0.492	203	1.01
12	7	High	52.59	96.67	0.17	0.477	203	1.01
17	7	High	54.23	76.56	0.188	0.509	203	1.01
15	7	High	55.3	85.31	0.168	0.486	203	1.01
29	7	High	53.47	98.82	0.18	0.471	203	1.01
19	7	High	52.79	106.35	0.148	0.484	203	1.01
7	7	High	50.29	91.94	0.166	0.493	203	1.01
10	7	High	53.46	85.28	0.172	0.499	203	1.01
24	7	High	51.82	74.8	0.171	0.502	203	1.01
24	7	High	51.27	74.8	0.171	0.502	203	1.01
11	7	High	52.19	105.96	0.178	0.492	203	1.01
24	7	High	49.86	74.8	0.171	0.502	203	1.01
24	7	High	49.93	74.8	0.171	0.502	203	1.01
24	7	High	49.93	74.8	0.171	0.502	203	1.01
12	7	High	51.23	96.67	0.17	0.477	203	1.01
2	7	High	51.87	103.88	0.161	0.471	203	1.01
20	7	High	55.49	89.72	0.177	0.517	203	1.01
19	7	High	56.62	106.35	0.148	0.484	203	1.01
6	7	High	52.32	111.35	0.165	0.468	203	1.01

24	7	High	52.06	74.8	0.171	0.502	203	1.01
10	7	High	51.92	85.28	0.172	0.499	203	1.01
24	7	High	51.7	74.8	0.171	0.502	203	1.01
11	7	High	54.52	105.96	0.178	0.492	203	1.01
10	7	High	50.52	85.28	0.172	0.499	203	1.01
29	7	High	48.55	98.82	0.18	0.471	203	1.01
7	7	High	47.34	91.94	0.166	0.493	203	1.01
15	7	High	53.74	85.31	0.168	0.486	203	1.01
12	7	High	51.14	96.67	0.17	0.477	203	1.01
6	7	High	48.86	111.35	0.165	0.468	203	1.01
6	7	High	56.01	111.35	0.165	0.468	203	1.01
5	7	High	53.32	97.7	0.163	0.517	203	1.01
24	7	High	48.29	74.8	0.171	0.502	203	1.01
29	7	High	51.1	98.82	0.18	0.471	203	1.01
24	7	High	50.46	74.8	0.171	0.502	203	1.01
4	7	High	52.6	83.82	0.176	0.457	203	1.01
3	7	High	51.23	93.13	0.146	0.466	203	1.01
4	7	High	48.5	83.82	0.176	0.457	203	1.01
7	7	High	49.19	91.94	0.166	0.493	203	1.01
28	7	High	57.46	118	0.166	0.527	203	1.01
24	7	High	46.71	74.8	0.171	0.502	203	1.01
1	7	High	42.3	88.13	0.173	0.483	203	1.01
5	7	High	52.81	97.7	0.163	0.517	203	1.01
14	7	High	54.44	110.71	0.181	0.467	203	1.01
7	7	High	50.96	91.94	0.166	0.493	203	1.01
24	7	High	45.39	74.8	0.171	0.502	203	1.01
17	7	High	51.4	76.56	0.188	0.509	203	1.01
29	7	High	52.59	98.82	0.18	0.471	203	1.01
19	7	High	53.66	106.35	0.148	0.484	203	1.01
5	7	High	54.28	97.7	0.163	0.517	203	1.01

15	7	High	52.34	85.31	0.168	0.486	203	1.01
15	7	High	55.48	85.31	0.168	0.486	203	1.01
7	7	High	51.78	91.94	0.166	0.493	203	1.01
11	7	High	46.17	105.96	0.178	0.492	203	1.01
23	7	High	48.07	76.25	0.155	0.484	203	1.01
15	7	High	49.18	85.31	0.168	0.486	203	1.01
12	7	High	52.65	96.67	0.17	0.477	203	1.01
27	9	High	54.4	82.79	0.175	0.432	196	0.406
23	9	High	49.16	76.25	0.155	0.484	196	0.406
10	9	High	51.76	85.28	0.172	0.499	196	0.406
18	9	High	54.16	117.5	0.192	0.51	196	0.406
11	9	High	57.4	105.96	0.178	0.492	196	0.406
4	9	High	55.91	83.82	0.176	0.457	196	0.406
11	9	High	55.7	105.96	0.178	0.492	196	0.406
14	9	High	55.88	110.71	0.181	0.467	196	0.406
4	9	High	56.27	83.82	0.176	0.457	196	0.406
30	9	High	52.35	75.77	0.172	0.498	196	0.406
2	9	High	54.82	103.88	0.161	0.471	196	0.406
30	9	High	47.91	75.77	0.172	0.498	196	0.406
12	9	High	58.1	96.67	0.17	0.477	196	0.406
17	9	High	50	76.56	0.188	0.509	196	0.406
17	9	High	58.92	76.56	0.188	0.509	196	0.406
11	9	High	56.4	105.96	0.178	0.492	196	0.406
22	9	High	53.34	81.3	0.132	0.491	196	0.406
14	9	High	62.73	110.71	0.181	0.467	196	0.406
12	9	High	57.93	96.67	0.17	0.477	196	0.406
19	9	High	59.7	106.35	0.148	0.484	196	0.406
14	9	High	58.24	110.71	0.181	0.467	196	0.406
18	9	High	56.77	117.5	0.192	0.51	196	0.406
16	9	High	58.29	122.22	0.18	0.519	196	0.406

2	9	High	53.13	103.88	0.161	0.471	196	0.406
19	9	High	56.52	106.35	0.148	0.484	196	0.406
26	9	High	58.89	125.1	0.173	0.459	196	0.406
1	9	High	50.86	88.13	0.173	0.483	196	0.406
4	9	High	54.76	83.82	0.176	0.457	196	0.406
10	9	High	52.51	85.28	0.172	0.499	196	0.406
15	9	High	54.11	85.31	0.168	0.486	196	0.406
11	9	High	54.6	105.96	0.178	0.492	196	0.406
1	9	High	56.06	88.13	0.173	0.483	196	0.406
30	9	High	50.61	75.77	0.172	0.498	196	0.406
20	9	High	57.67	89.72	0.177	0.517	196	0.406
13	9	High	58.5	77.78	0.139	0.507	196	0.406
26	9	High	59.71	125.1	0.173	0.459	196	0.406
26	9	High	55.36	125.1	0.173	0.459	196	0.406
12	9	High	54.63	96.67	0.17	0.477	196	0.406
12	9	High	54.57	96.67	0.17	0.477	196	0.406
11	9	High	56.4	105.96	0.178	0.492	196	0.406
27	9	High	54.1	82.79	0.175	0.432	196	0.406
19	9	High	58.37	106.35	0.148	0.484	196	0.406
19	9	High	57.83	106.35	0.148	0.484	196	0.406
8	9	High	59.21	95.41	0.179	0.488	196	0.406
1	9	High	55.04	88.13	0.173	0.483	196	0.406
13	9	High	56.23	77.78	0.139	0.507	196	0.406
19	9	High	56.24	106.35	0.148	0.484	196	0.406
1	9	High	57.9	88.13	0.173	0.483	196	0.406
26	9	High	60.35	125.1	0.173	0.459	196	0.406
19	9	High	56.4	106.35	0.148	0.484	196	0.406
10	9	High	53.33	85.28	0.172	0.499	196	0.406
13	9	High	56.08	77.78	0.139	0.507	196	0.406
8	9	High	55.61	95.41	0.179	0.488	196	0.406

12	9	High	56.86	96.67	0.17	0.477	196	0.406
15	9	High	55.26	85.31	0.168	0.486	196	0.406
3	9	High	53.37	93.13	0.146	0.466	196	0.406
5	9	High	55.54	97.7	0.163	0.517	196	0.406
18	9	High	58.03	117.5	0.192	0.51	196	0.406
6	9	High	60.02	111.35	0.165	0.468	196	0.406
30	9	High	51.2	75.77	0.172	0.498	196	0.406
30	9	High	49.89	75.77	0.172	0.498	196	0.406
18	9	High	54.85	117.5	0.192	0.51	196	0.406
29	9	High	52.46	98.82	0.18	0.471	196	0.406
9	9	High	55.65	80.99	0.172	0.484	196	0.406
23	9	High	48.9	76.25	0.155	0.484	196	0.406
18	9	High	59.26	117.5	0.192	0.51	196	0.406
9	9	High	55.19	80.99	0.172	0.484	196	0.406
18	9	High	58	117.5	0.192	0.51	196	0.406
23	9	High	52.21	76.25	0.155	0.484	196	0.406
26	9	High	55.15	125.1	0.173	0.459	196	0.406
27	9	High	56.12	82.79	0.175	0.432	196	0.406
14	9	High	61.79	110.71	0.181	0.467	196	0.406
11	9	High	53.45	105.96	0.178	0.492	196	0.406
10	9	High	55.54	85.28	0.172	0.499	196	0.406
13	9	High	54.72	77.78	0.139	0.507	196	0.406
13	9	High	55.99	77.78	0.139	0.507	196	0.406
23	9	High	50.93	76.25	0.155	0.484	196	0.406
11	9	High	52.55	105.96	0.178	0.492	196	0.406
4	9	High	55.61	83.82	0.176	0.457	196	0.406
30	9	High	50.74	75.77	0.172	0.498	196	0.406
18	9	High	58.36	117.5	0.192	0.51	196	0.406
20	9	High	59.12	89.72	0.177	0.517	196	0.406
1	9	High	53.23	88.13	0.173	0.483	196	0.406

23	9	High	47.73	76.25	0.155	0.484	196	0.406
27	9	High	57.57	82.79	0.175	0.432	196	0.406
19	9	High	60.19	106.35	0.148	0.484	196	0.406
17	9	High	55.29	76.56	0.188	0.509	196	0.406
18	9	High	58.72	117.5	0.192	0.51	196	0.406
27	9	High	54.25	82.79	0.175	0.432	196	0.406
14	9	High	55.54	110.71	0.181	0.467	196	0.406
17	9	High	58.83	76.56	0.188	0.509	196	0.406
5	9	High	57.27	97.7	0.163	0.517	196	0.406
6	9	High	57.37	111.35	0.165	0.468	196	0.406
21	9	High	59.26	121.06	0.173	0.494	196	0.406
20	9	High	57.34	89.72	0.177	0.517	196	0.406
4	9	High	56.56	83.82	0.176	0.457	196	0.406
13	9	High	47.63	77.78	0.139	0.507	196	0.406
17	9	High	56.58	76.56	0.188	0.509	196	0.406
12	9	High	54.62	96.67	0.17	0.477	196	0.406
10	9	High	53.68	85.28	0.172	0.499	196	0.406
18	9	High	60.05	117.5	0.192	0.51	196	0.406
1	9	High	54.16	88.13	0.173	0.483	196	0.406
17	9	High	54.25	76.56	0.188	0.509	196	0.406
9	9	High	49.64	80.99	0.172	0.484	196	0.406
18	9	High	62.75	117.5	0.192	0.51	196	0.406
30	9	High	50.84	75.77	0.172	0.498	196	0.406
12	9	High	63.32	96.67	0.17	0.477	196	0.406
30	9	High	51.35	75.77	0.172	0.498	196	0.406
4	9	High	53.99	83.82	0.176	0.457	196	0.406
12	9	High	54.59	96.67	0.17	0.477	196	0.406
19	9	High	60.72	106.35	0.148	0.484	196	0.406
27	9	High	57.76	82.79	0.175	0.432	196	0.406
1	9	High	53.02	88.13	0.173	0.483	196	0.406

12	9	High	52.22	96.67	0.17	0.477	196	0.406
13	9	High	54.65	77.78	0.139	0.507	196	0.406
7	9	High	57.22	91.94	0.166	0.493	196	0.406
11	9	High	55.01	105.96	0.178	0.492	196	0.406
23	9	High	53.27	76.25	0.155	0.484	196	0.406
19	9	High	53.9	106.35	0.148	0.484	196	0.406
7	9	High	53.5	91.94	0.166	0.493	196	0.406
2	9	High	54.54	103.88	0.161	0.471	196	0.406
20	9	High	56.06	89.72	0.177	0.517	196	0.406
4	9	High	53.4	83.82	0.176	0.457	196	0.406
8	10	High	54.14	95.41	0.179	0.488	203	0.845
24	10	High	49.85	74.8	0.171	0.502	203	0.845
10	10	High	52.9	85.28	0.172	0.499	203	0.845
12	10	High	53.19	96.67	0.17	0.477	203	0.845
10	10	High	54.45	85.28	0.172	0.499	203	0.845
12	10	High	53.5	96.67	0.17	0.477	204	0.845
7	10	High	53.98	91.94	0.166	0.493	204	0.845
24	10	High	48.1	74.8	0.171	0.502	204	0.845
24	10	High	47.42	74.8	0.171	0.502	203	0.845
5	10	High	58.67	97.7	0.163	0.517	204	0.845
28	10	High	56.14	118	0.166	0.527	203	0.845
24	10	High	47.82	74.8	0.171	0.502	203	0.845
24	10	High	45.2	74.8	0.171	0.502	203	0.845
10	10	High	52.07	85.28	0.172	0.499	203	0.845
7	10	High	57.13	91.94	0.166	0.493	203	0.845
11	10	High	52.17	105.96	0.178	0.492	204	0.845
4	10	High	53.81	83.82	0.176	0.457	204	0.845
24	10	High	53	74.8	0.171	0.502	203	0.845
29	10	High	54.41	98.82	0.18	0.471	204	0.845
11	10	High	55.99	105.96	0.178	0.492	203	0.845

28	10	High	57.66	118	0.166	0.527	203	0.845
11	10	High	54.78	105.96	0.178	0.492	203	0.845
29	10	High	54.56	98.82	0.18	0.471	204	0.845
7	10	High	45.99	91.94	0.166	0.493	203	0.845
24	10	High	48.14	74.8	0.171	0.502	203	0.845
11	10	High	59.92	105.96	0.178	0.492	204	0.845
5	10	High	52.8	97.7	0.163	0.517	203	0.845
12	10	High	55.47	96.67	0.17	0.477	203	0.845
16	10	High	53.13	122.22	0.18	0.519	203	0.845
29	10	High	49.41	98.82	0.18	0.471	204	0.845
7	10	High	54.63	91.94	0.166	0.493	203	0.845
12	10	High	55.07	96.67	0.17	0.477	203	0.845
24	10	High	52.74	74.8	0.171	0.502	203	0.845
13	10	High	54.51	77.78	0.139	0.507	203	0.845
20	10	High	60.14	89.72	0.177	0.517	203	0.845
20	10	High	52.56	89.72	0.177	0.517	204	0.845
3	10	High	51.85	93.13	0.146	0.466	203	0.845
24	10	High	50.01	74.8	0.171	0.502	204	0.845
15	10	High	50.68	85.31	0.168	0.486	204	0.845
24	10	High	50.72	74.8	0.171	0.502	204	0.845
29	10	High	55.07	98.82	0.18	0.471	203	0.845
1	10	High	43.22	88.13	0.173	0.483	203	0.845
5	10	High	56.5	97.7	0.163	0.517	203	0.845
5	10	High	57.18	97.7	0.163	0.517	203	0.845
5	10	High	53.79	97.7	0.163	0.517	203	0.845
24	10	High	51.07	74.8	0.171	0.502	203	0.845
3	10	High	40.98	93.13	0.146	0.466	203	0.845
4	10	High	50.68	83.82	0.176	0.457	203	0.845
16	10	High	56.36	122.22	0.18	0.519	203	0.845
27	10	High	51.25	82.79	0.175	0.432	203	0.845

14	10	High	54.81	110.71	0.181	0.467	203	0.845
20	10	High	57.28	89.72	0.177	0.517	203	0.845
20	10	High	56.06	89.72	0.177	0.517	203	0.845
26	10	High	60.26	125.1	0.173	0.459	203	0.845
2	10	High	53.19	103.88	0.161	0.471	203	0.845
14	10	High	59.72	110.71	0.181	0.467	203	0.845
12	10	High	48.25	96.67	0.17	0.477	204	0.845
12	10	High	52.12	96.67	0.17	0.477	203	0.845
29	10	High	51.79	98.82	0.18	0.471	203	0.845
19	10	High	53.39	106.35	0.148	0.484	203	0.845
24	10	High	52.76	74.8	0.171	0.502	203	0.845
10	10	High	56.44	85.28	0.172	0.499	204	0.845
29	10	High	55.45	98.82	0.18	0.471	203	0.845
10	10	High	50.8	85.28	0.172	0.499	203	0.845
15	10	High	52.36	85.31	0.168	0.486	203	0.845
24	10	High	47.71	74.8	0.171	0.502	203	0.845
24	10	High	52.52	74.8	0.171	0.502	203	0.845
15	10	High	54.67	85.31	0.168	0.486	204	0.845
1	10	High	54.07	88.13	0.173	0.483	203	0.845
7	10	High	54.49	91.94	0.166	0.493	203	0.845
29	10	High	56.78	98.82	0.18	0.471	203	0.845
24	10	High	53.63	74.8	0.171	0.502	203	0.845
12	10	High	52.81	96.67	0.17	0.477	204	0.845
9	10	High	49.74	80.99	0.172	0.484	203	0.845
15	10	High	55.6	85.31	0.168	0.486	204	0.845
10	10	High	50.36	85.28	0.172	0.499	203	0.845
29	10	High	52.2	98.82	0.18	0.471	203	0.845
12	10	High	52.04	96.67	0.17	0.477	203	0.845
3	10	High	51.66	93.13	0.146	0.466	203	0.845
17	10	High	52.19	76.56	0.188	0.509	203	0.845

5	10	High	53.15	97.7	0.163	0.517	203	0.845
20	10	High	56.47	89.72	0.177	0.517	203	0.845
29	10	High	52.79	98.82	0.18	0.471	203	0.845
24	10	High	48.55	74.8	0.171	0.502	204	0.845
27	10	High	56.39	82.79	0.175	0.432	203	0.845
7	10	High	53.66	91.94	0.166	0.493	203	0.845
28	10	High	55.99	118	0.166	0.527	203	0.845
15	10	High	52.93	85.31	0.168	0.486	204	0.845
29	10	High	53.44	98.82	0.18	0.471	203	0.845
10	10	High	49.97	85.28	0.172	0.499	203	0.845
19	10	High	57.27	106.35	0.148	0.484	203	0.845
12	10	High	51.27	96.67	0.17	0.477	203	0.845
17	10	High	49.13	76.56	0.188	0.509	203	0.845
14	10	High	56.09	110.71	0.181	0.467	204	0.845
12	10	High	53.29	96.67	0.17	0.477	204	0.845
19	10	High	51.51	106.35	0.148	0.484	203	0.845
5	10	High	52.73	97.7	0.163	0.517	203	0.845
15	10	High	54.21	85.31	0.168	0.486	203	0.845
4	10	High	49.02	83.82	0.176	0.457	204	0.845
3	10	High	51.94	93.13	0.146	0.466	203	0.845
27	10	High	48.73	82.79	0.175	0.432	203	0.845
29	10	High	55.96	98.82	0.18	0.471	203	0.845
19	10	High	54.43	106.35	0.148	0.484	203	0.845
27	10	High	52.69	82.79	0.175	0.432	203	0.845
10	10	High	48.37	85.28	0.172	0.499	203	0.845
15	10	High	52.12	85.31	0.168	0.486	203	0.845
10	10	High	45.8	85.28	0.172	0.499	203	0.845
7	10	High	54.97	91.94	0.166	0.493	203	0.845
6	10	High	55.33	111.35	0.165	0.468	204	0.845
27	10	High	53.11	82.79	0.175	0.432	203	0.845

7	10	High	48.24	91.94	0.166	0.493	203	0.845
12	10	High	52.76	96.67	0.17	0.477	203	0.845
24	10	High	52.6	74.8	0.171	0.502	203	0.845
12	10	High	54.91	96.67	0.17	0.477	203	0.845
7	10	High	51.24	91.94	0.166	0.493	203	0.845
29	10	High	54.41	98.82	0.18	0.471	203	0.845
28	10	High	55.77	118	0.166	0.527	204	0.845
20	10	High	54.72	89.72	0.177	0.517	203	0.845
17	10	High	56.07	76.56	0.188	0.509	203	0.845
7	10	High	54.48	91.94	0.166	0.493	203	0.845
20	10	High	61.29	89.72	0.177	0.517	203	0.845
10	10	High	48.89	85.28	0.172	0.499	204	0.845
5	10	High	59.06	97.7	0.163	0.517	203	0.845
9	10	High	55.56	80.99	0.172	0.484	204	0.845
19	10	High	51.5	106.35	0.148	0.484	204	0.845
15	10	High	54.24	85.31	0.168	0.486	204	0.845
9	10	High	54.39	80.99	0.172	0.484	204	0.845
16	10	High	54.98	122.22	0.18	0.519	203	0.845
12	10	High	53.97	96.67	0.17	0.477	203	0.845
12	10	High	55.54	96.67	0.17	0.477	203	0.845
5	10	High	53.01	97.7	0.163	0.517	203	0.845

94
